# Human DNA topoisomerase I poisoning causes R loop–mediated genome instability attenuated by transcription factor IIS

**DOI:** 10.1126/sciadv.adm8196

**Published:** 2024-05-24

**Authors:** Renée C. Duardo, Jessica Marinello, Marco Russo, Sara Morelli, Simona Pepe, Federico Guerra, Belén Gómez-González, Andrés Aguilera, Giovanni Capranico

**Affiliations:** ^1^ Department of Pharmacy and Biotechnology, Alma Mater Studiorum–University of Bologna, via Selmi 3, 40126, Bologna, Italy.; ^2^ Centro Andaluz de Biología Molecular y Medicina Regenerativa—CABIMER, Universidad de Sevilla–CSIC, Calle Américo Vespucio 24, 41092 Seville, Spain.; ^3^ Departamento de Genetica, Facultad de Biología, Universidad de Sevilla, 41012 Seville, Spain.

## Abstract

DNA topoisomerase I can contribute to cancer genome instability. During catalytic activity, topoisomerase I forms a transient intermediate, topoisomerase I–DNA cleavage complex (Top1cc) to allow strand rotation and duplex relaxation, which can lead to elevated levels of DNA-RNA hybrids and micronuclei. To comprehend the underlying mechanisms, we have integrated genomic data of Top1cc-triggered hybrids and DNA double-strand breaks (DSBs) shortly after Top1cc induction, revealing that Top1ccs increase hybrid levels with different mechanisms. DSBs are at highly transcribed genes in early replicating initiation zones and overlap with hybrids downstream of accumulated RNA polymerase II (RNAPII) at gene 5′-ends. A transcription factor IIS mutant impairing transcription elongation further increased RNAPII accumulation likely due to backtracking. Moreover, Top1ccs can trigger micronuclei when occurring during late G_1_ or early/mid S, but not during late S. As micronuclei and transcription-replication conflicts are attenuated by transcription factor IIS, our results support a role of RNAPII arrest in Top1cc-induced transcription-replication conflicts leading to DSBs and micronuclei.

## Teaser

Integrating maps of DNA-RNA hybrid and DNA double-strand cuts determines genomic sites of transcription/replication conflicts.

## INTRODUCTION

DNA topoisomerases are a family of essential nuclear enzymes that regulate DNA topology, chromatin structures, and basic processes such as transcription and replication (*1*). DNA topoisomerase I (Top1) couples DNA strand cleavage-ligation reaction with the rotation of the cut strand around the other, therefore reducing the torsional tension of DNA duplexes (*2*, *3*). During the catalytic cycle, a reaction intermediate called Top1-DNA cleavage complex (Top1cc) forms, wherein Top1 is covalently linked to the 3′-end of the cut strand. However, under certain circumstances, Top1ccs can trigger genome instability (*4*). Top1ccs can be stabilized by several factors, including U.S. Food and Drug Administration–approved antitumor Top1 poisons, such as camptothecin (CPT) analogs. CPT selectively targets Top1 in living cells (*2*, *3*), leading to a rapid (2 to 5 min) increase in Top1ccs (*5*, *6*). Within minutes, they affect RNA and DNA synthesis markedly (*2*, *3*), impair DNA transactions and epigenomic features (*5*–*10*), and activate ubiquitin-dependent Top1 degradation (*3*, *11*, *12*). Moreover, CPT-induced Top1ccs increase transcription-replication conflicts (TRCs) (*13*–*16*) and DNA double-strand breaks (DSBs) (*3*, *4*, *17*, *18*). The generation of DSBs may be due to replication run off at Top1cc sites (*19*, *20*) or to the activity of endonucleases at stalled forks (*21*–*25*). TRCs occur more often in cancer than normal cells due to high oncogene-induced transcription rates and can thus be at the basis of high genome instability in cancer (*13*–*15*).

Top1ccs can also occur in unperturbed cells as they can arise when the DNA substrate is damaged (*4*, *26*). Top1ccs were shown to be a general pathogenic feature of neurodegenerative disorders derived from mutations of DNA repair factors (*27*). A mutation of Tyrosyl-DNA phosphodiesterase 1 (TDP1), a specific repair factor of Top1ccs, can cause a rare type of ataxia spinocerebellar ataxia with axonal neuropathy 1 (SCAN1) (*28*) characterized by cell hypersensitivity to CPT (*29*). Moreover, Top1 can introduce short indels in the genome at sites of misincorporated ribonucleotides (*30*, *31*). Thus, impaired Top1 activity can be dangerous to cell life and differentiation when nuclear Top1cc levels become elevated because of exogenous or endogenous factors. However, the mechanism elicited by Top1ccs is not yet fully understood.

Top1ccs can effectively induce TRCs with a yet undefined R loop–mediated mechanism. R loops are three-strand nucleic acid structures containing a hybrid DNA:RNA duplex (*15*, *32*). Top1 poisons cause a transient increase in R loops in cancer cells (*5*, *18*, *33*, *34*). However, genomic R loop maps are not yet available upon Top1cc increase. Recently, we showed that micronuclei induced by Top1ccs are dependent on R loops (*18*). In addition, mutations of ribonuclease (RNase) H2, an endonuclease specifically targeting RNA strands of hybrid duplexes, can cause the human Aicardi-Goutières syndrome and an increase in micronuclei triggering a persistent inflammatory activation, likely at the basis of the syndrome (*35*–*37*). However, the mechanism of hybrid-dependent TRCs, DNA cleavage, and micronucleus formation remains largely unknown in human cancer cells.

In this study, we uncover a main mechanism by which Top1ccs induce R loops and TRCs. By determining the genomic maps of DNA-RNA hybrid and DSB loci upon Top1cc induction, we provide evidence for the role of transcription elongation factor IIS (TFIIS) in attenuating TRCs and genome instability. Moreover, analysis of cell cycle phase–dependent triggering of micronuclei allowed us to show that Top1ccs affect genome stability specifically during late G_1_/early S phases. Our findings establish an early replication-specific and transcription-dependent mechanism of genome instability elicited by Top1ccs and provide the main genomic sites of transcription/replication conflicts (TCRs) induced by Top1ccs. These findings can aid in the development of strategies for neurodegeneration prevention and cancer treatment.

## RESULTS

### Top1ccs induce stable and transient hybrid gains at highly transcribed genes

**Fig. 1.**
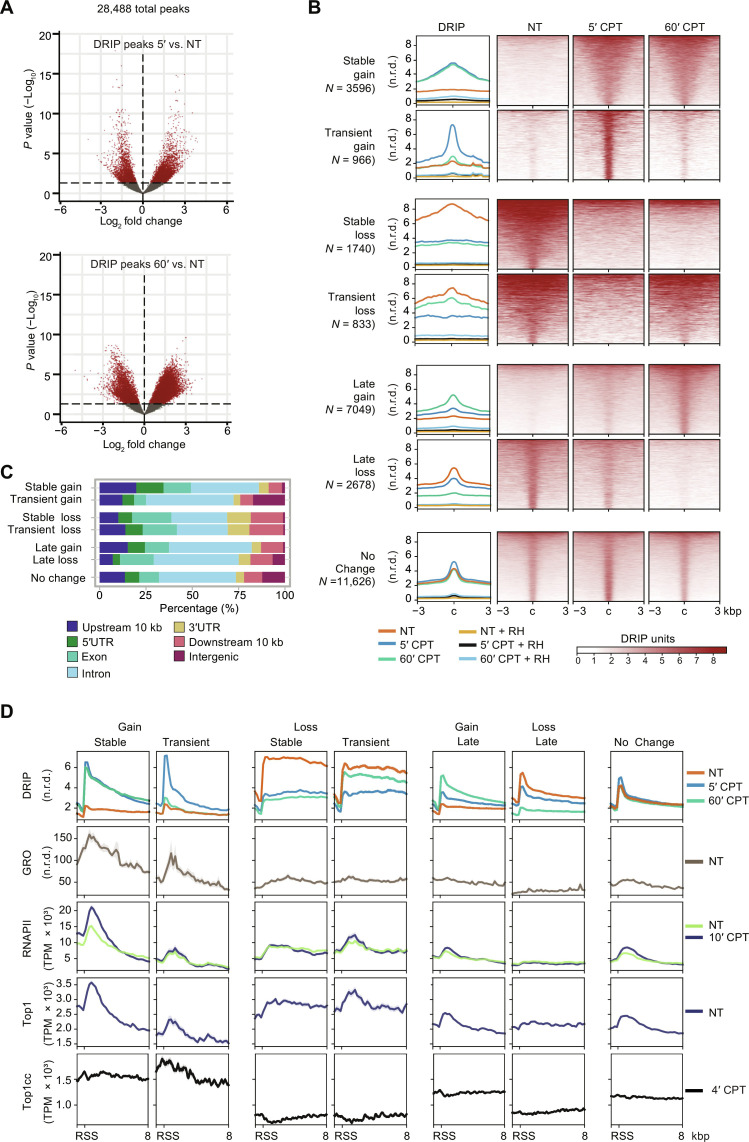
Top1ccs induce dynamic changes of hybrid levels at specific genomic sites in HCT116 cells. (**A**) Volcano plots of differential analyses of DRIP-seq signals between cells treated with CPT for 5 min (5′ CPT) versus untreated cells (NT) (top) and cells treated with CPT for 60 min (60’ CPT) versus NT (bottom). Each dot represents a DRIP-seq peak. Axis values are log_2_(fold changes) of DRIP-seq levels (*x* axis) and −log_10_(*P* values) of limma test (*y* axis). Red dots indicate peaks with *P* value less than 0.01, shown as a dashed line. (**B**) Metaplots (left) and heatmap (right) of DRIP-seq normalized read density (n.r.d.) in seven R loop categories distinguished by different kinetics. Line colors of metaplots correspond to the means of two biological replicates as reported in the legend. Heatmap colors represent DRIP levels as in legend. In both metaplots and heatmaps, “c” indicates DRIP peak center in a window of + or –3 kilo–base pairs (kbp). RH indicates DRIP samples treated with RNase H1 before immunoprecipitation. (**C**) Fractions of DRIP peaks over genomic features, as shown in the legend. (**D**) Metaplots of DRIP-seq (5- and 60-min treatment with CPT), GRO-seq (*38*), RNAPII ChIP-seq (10-min treatment with CPT) (*6*), Top1 ChIP-seq (*6*), and Top1cc-seq (4-min treatment with CPT) (*6*) normalized levels for each R loop kinetic category. RSS is the start site of R loop peaks. The graph is in a window of −1/+8 kbp with respect to RSS. Line colors as in legend.

To establish the role of R loops in genome instability by Top1ccs, we first determined the genomic maps of DNA-RNA hybrids. Cell treatment with CPT increases Top1ccs at the highest levels within 2 to 5 min (*5*, *6*) and DNA-RNA hybrid levels after 5 to 10 min (*5*, *18*) in human cancer cells (fig. S1, A and B). Thus, we determined the hybrid maps immediately upon Top1cc increase, after 5-min CPT treatment, compared with a longer treatment (60 min). After read normalization (fig. S1, C and D, and table S1), the results showed that Top1ccs induced either a reduction (loss) or an increase (gain) in hybrid levels at thousands of loci in a time-dependent manner (Fig. 1, A and B). Hybrid peaks were then split into seven categories based on their kinetics: Stable gains or losses showed hybrid changes found at 5 min that persisted at 60 min; transient gains and losses showed hybrid changes found at 5 min that did not persist at 60 min; late gains and losses showed hybrid changes detected only at 60 min; and, last, no change hybrids that did not show any significant alteration at either times (Fig. 1, A and B). Thus, Top1ccs can dynamically alter nuclear hybrid levels in either direction.

Top1cc-altered hybrid peaks show size differences (fig. S1E) and were largely localized along genes, particularly at introns (Fig. 1C and fig. S1F). Specifically, stable hybrid gains and losses are enriched at 5′ untranslated region (5′UTR)/upstream and 3′UTR/downstream regions, respectively (Fig. 1C and fig. S1F). Gene size was slightly different among hybrid categories (fig. S1G). As determined by global run-on sequencing (GRO-seq) data (*38*), all Top1cc-altered hybrids, except late loss peaks, were in transcribed regions, and stable gain peaks showed a strong association with highest transcription rates (Fig. 1D). Stable hybrid gain peaks were found in open chromatin, as determined by H3K4me3 and H3K27ac histone marks (fig. S1H). Gain but not loss peaks were closer to lamina-associated domains (fig. S1I), whereas all hybrid peaks were similarly distant from heterochromatin sites, as determined by H3K9me3 (fig. S1J) [histone markers are from ENCODE database of HCT116 cells (*39*)]. Thus, we conclude that hybrid gains, in particular, stable ones, are at highly transcribed genes in an open chromatin context that can favor R loop formation.

**Fig. 2.**
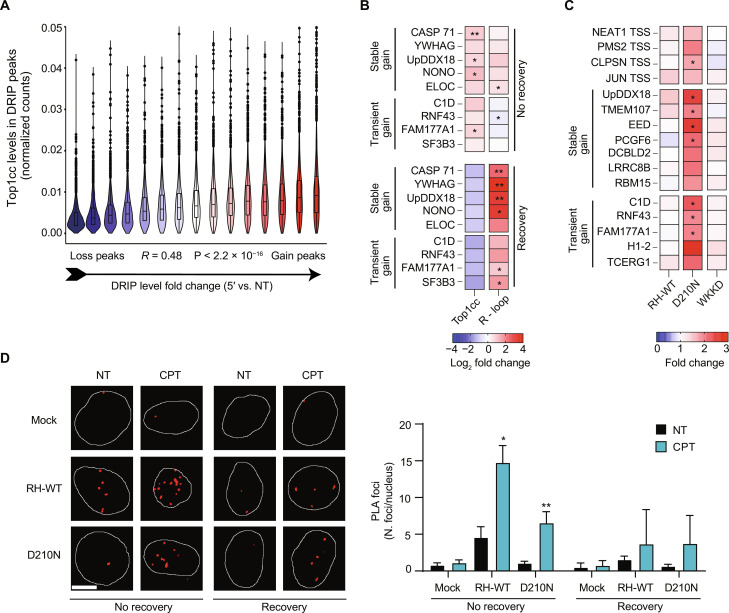
Top1ccs are close to hybrid gain peaks. (**A**) Violin plot of Top1cc levels (*y* axis), computed using Top1cc-seq experiment, in R loop peaks divided in 15 groups on the base of 5 min-CPT versus untreated (NT) log_2_(fold change) of DRIP-seq levels (*x* axis). Spearman correlation coefficient and relative *P* values are reported in the graph. (**B**) Heatmap showing Top1cc and hybrid levels at stable and transient gain hybrid peaks in 5-min CPT–treated samples. Cells were lysed directly after drug administration (no recovery) or after 15 min of recovery in drug-free medium (recovery). DNA enrichment over input after K^+^/SDS (Top1cc) and DRIP (R loop) assays was quantified by real-time PCR and reported as mean fold change (log_2_) of treated versus untreated samples. DRIP enrichment over input was normalized on pFC53 plasmid spike-in [R loop fragment (RF) amplicon]. At least three biological replicates are reported. (**C**) Heatmap showing R-ChIP analysis at stable and transient gain hybrid peaks in 5-min CPT–treated samples. Transcription start site (TSS) of NEAT (NEAT1 TSS), PMS2 (PMS2 TSS), CLPSN (CLPSN TSS), and JUN (JUN TSS) genes were used as R-ChIP–positive controls (*43*). DNA enrichment over input was quantified by real-time PCR and reported as mean fold change (log_2_) of treated versus untreated samples. At least three biological replicates are reported. (**D**) Representative images and PLA focus analysis of untreated and 5-min CPT–treated samples. PLA of Top1cc (clone1.1A, #MABE1084) with V5-tagged RNase H1 (#ab15828) was performed. Cells were fixed directly after drug administration (no recovery) or after 15 min of recovery in drug-free medium (recovery). Each bar represents the mean values ± SEM from two biological replicates. The average number of cells analyzed is 200. For all data, statistical significance was performed on fold change values with one-tailed ratio paired *t* test. *P < 0.05; **P < 0.01.

Consistently, significant levels of RNA polymerase II (RNAPII) and Top1 [datasets from (*6*)] were found at altered DNA-RNA hybrid peaks with highest levels found at stable gain peaks (Fig. 1D and fig. S2A). Instead, Top1ccs [datasets from (*6*)] were at the highest levels at stable and transient gain peaks (Fig. 1D and fig. S2, A and B). Consistently, considering all hybrid peaks at 5 min, Top1cc levels showed a linear association with the gain rate with respect to untreated cells (Fig. 2A), strongly supporting that hybrid transient and stable gains are directly induced by Top1ccs. We notice that, in untreated cells, transient and stable loss peaks showed hybrid levels higher than gain peaks and high Top1 levels but undetectable Top1ccs (Fig. 1D and fig. S2A), suggesting a very low Top1 activity. Thus, a high Top1 activity is likely critical to keep low hybrid levels in untreated cells at gain peak regions, which showed high transcription. This is consistent with a major requirement of Top1 activity at highly transcribed genes that, in turn, would prevent hybrid formation.

### Top1ccs are close to hybrid gain peaks

To better define the relation between Top1cc and DNA-RNA hybrid gains, we measured the amount of Top1ccs by Top1cc K^+^/SDS precipitation (*40*, *41*) and hybrid levels by DNA/RNA immunoprecipitation (DRIP)–quantitative polymerase chain reaction (qPCR) at peaks showing stable and transient gains. Top1ccs accumulated at all these sites (Fig. 2B), but hybrids were barely detected when cell lysis was performed immediately after treatment (Fig. 2B). Conversely, a recovery time of 15 min before cell lysis allowed Top1cc reversion (Fig. 2B), as expected (*2*, *3*), and detection of high hybrid levels (Fig. 2B). A recovery step of ~15 min was similarly included in DRIP sequencing (DRIP-seq) protocol. Stable hybrid gains could still be detected although to a significantly lower extent, whereas transient gains were fully lost with no recovery (Fig. 2B), indicating that stable and transient gains can correspond to structurally different R loops. Together, the results show that increased hybrids can be destabilized by the presence of Top1ccs during the DRIP protocol, in agreement with the observation that DNA nicks can affect hybrid stability in vitro (*42*). Thus, hybrids can be lost during the DNA extraction step of the DRIP protocol if lysis is performed immediately upon drug removal. To confirm this hypothesis, we measured hybrid levels with the R-loop-chromatin immunoprecipitation (R-ChIP) technique, which does not require DNA extraction (*43*). We checked R-ChIP efficiency with positive and negative loci (fig. S2C) (*43*). The results demonstrated that hybrids increased at all gain peak sites immediately upon Top1cc induction (Fig. 2C), supporting that Top1ccs are close enough to gain peaks to affect hybrid formation in living cells and during the DRIP protocol. Proximity ligation assay (PLA) results further supported that Top1ccs are close to hybrids, as inferred from positive signals obtained with specific antibodies against Top1ccs or V5-tagged RNase H1 (Fig. 2D). Thus, hybrid gains are caused locally by Top1ccs at highly transcribed genes where Top1 activity is high to maintain a low level of R loop structures and a high RNAPII elongation rate (*6*, *44*). As Top1cc induction by CPT increases negative supercoils at promoters of transcribed genes (*10*), high R loop levels can be due to increased unwinding tension, particularly at 5′-ends of highly transcribed genes. However, other models are possible, particularly along the gene body, as Top1ccs may provide a free DNA end to allow DNA-RNA annealing (*32*).

### Stable hybrid gains are downstream of RNAPII peaks, enhanced by a dominant negative TFIIS mutation

**Fig. 3.**
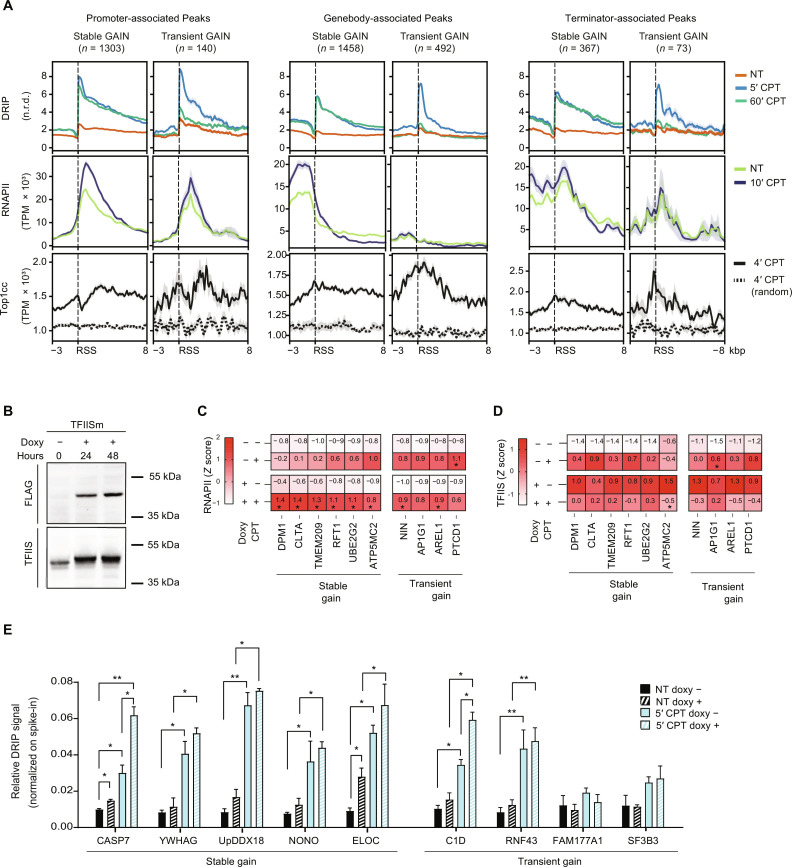
Stable hybrid gains are downstream of RNAPII peaks. (**A**) Metaplots of DRIP-seq, RNAPII ChIP-seq, and Top1cc-seq normalized levels at stable and transient R loop gain divided in promoter-associated (up to −10 kbp from promoter and 5′UTR), gene body–associated (located in exon and intron) and terminator-associated (located in 3′UTR and downstream 10 kbp) regions of genes. Dashed lines in Top1cc-seq metaplots show background levels of Top1cc after R loop gain randomization. RSS represents the R loop peak start site, in a window of −1/+8 kbp. Line colors as in legend. (**B**) Expression by Western blot of the dominant negative TFIISm upon doxycycline (doxy) exposure (+) in HEK293 cells. (**C** and **D**) Heatmaps showing RNAPII and TFIIS levels at selected loci, respectively, determined by ChIP (antibodies sc-47701 and ab185947, respectively). The tested loci overlap with Top1cc-induced RNAPII accumulation and the indicated hybrid gain regions, which are between 300 and 500 bp downstream of the TSS of the indicated genes. HEK293 cells were treated with CPT for 10 min. TFIISm was induced for 48 hours with doxycycline. DNA enrichment over input was quantified by real-time PCR and reported as *Z* score (shown in each cell). Data are from at least three biological replicates. Statistical significance was calculated by comparing treated over nontreated samples using multiple unpaired *t* test. (**E**) R loop levels determined by DRIP-qPCR at indicated loci in TFIISm induced and uninduced HEK293 cells. DRIP enrichment over input was normalized on pFC53 plasmid spike-in (RF amplicon). Each bar represents mean values ± SEM of at least three biological replicates. Statistical tests were performed with one-tailed ratio paired *t* test. In all panels, *P < 0.05; **P < 0.01.

Metaplot analyses of aligned hybrid regions showed a marked increase in RNAPII levels upon Top1cc induction at sites with stable hybrid gains, but not at other regions (Fig. 1D), suggesting a role for RNAPII elongation in stable hybrid increase. Thus, we next compared the relative orientation of RNAPII and Top1cc levels and hybrid regions for transient and stable hybrid gains (Fig. 3A and fig. S3A). Splitting hybrids based on genic location, we found that while levels of Top1cc in transient and stable hybrid regions are similar in each peak category, transient hybrid gains peaked upstream of accumulated RNAPII for promoter- and terminator-associated hybrids (Fig. 3A and fig. S3A), as expected for nascent RNA hybridizing to the template behind the RNAPII (*32*, *45*). In the case of gene body-associated transient gains, a well-defined RNAPII peak was not detected (Fig. 3A). However, for stable hybrid gains at gene bodies, RNAPII peaks were fully upstream of hybrids (Fig. 3A, middle) and at gene termination regions, RNAPII peaks were, in part, upstream of the hybrids for stable gains (Fig. 3A, right). RNAPII increased in Top1cc induction mainly upstream of hybrids (gene body and termination regions) or at the downstream side of peaks (promoters) (Fig. 3A and fig. S3A). Thus, Top1ccs can induce hybrid gains that are differently located relative to RNAPII peaks.

Together, our results raise the question of whether most of stable hybrid gains correspond to R loops downstream of RNAPII, possibly following RNAPII backtracking (fig. S3B, top) (*23*), or, alternatively, RNAPII peaks upstream of stable hybrids may reflect stalled RNA polymerases behind a hybrid formed by the nascent RNA of another RNAPII downstream (fig. S3B, bottom). To distinguish between these two possibilities, we studied the effects of TFIIS as this factor can bind to backtracked, arrested RNAPIIs to restart transcription elongation by transcript cleavage and has been reported to prevent R loop downstream of it (*46*). Therefore, we expressed a dominant-negative TFIIS mutant (TFIISm; D290A and E291A) in human embryonic kidney (HEK) 293T cells (*46*) (Fig. 3B and fig. S3C) and measured its effects on RNAPII levels at sites of Top1cc-induced RNAPII accumulation by ChIP (fig. S3D). The results confirmed that Top1ccs induced an accumulation of RNAPII at the tested sites located at 300 to 500 base pairs (bp) downstream of the transcription start site (TSS) of genes with stable or transient hybrid gains (Fig. 3C and fig. S3D), but not at silent genes or untranscribed sites (fig. S3D).

Top1ccs induced a higher accumulation of RNAPII at almost all stable, but not transient, hybrid gains upon TFIISm expression (Fig. 3C), showing that RNAPII accumulation can be attenuated by wild-type (WT) TFIIS. Using an antibody against the WT factor, TFIIS accumulated at all the tested sites upon TFIISm expression in CPT-untreated cells (Fig. 3D and fig. S3E), in agreement with published data showing higher recruitment of TFIIS within 1000 bp from TSS in the presence of expressed TFIISm [see figure 1F in (*47*)]. Top1ccs induced higher TFIIS levels in cells not expressing TFIISm, whereas they decreased TFIIS levels upon TFIISm expression (Fig. 3D and fig. S3E). In addition, TFIISm expression increased hybrid levels markedly at stable gains, but very slightly at transient gains, either in untreated or CPT-treated cells (Fig. 3E and fig. S3F), suggesting that WT TFIIS activity can reduce stable Top1cc-induced R loops. Thus, accumulation of RNAPII or WT TFIIS at sites of Top1ccs following TFIISm expression or CPT treatment, respectively, shows that transcript cleavage by WT TFIIS can restart transcription elongation and reduce Top1-induced RNAPII accumulation and R loop levels at genes with stable hybrid gains, suggesting a role for RNAPII backtracking in R loop increase.

### Top1ccs cause DSBs downstream of accumulated RNAPII at highly transcribed genes

Next, to get insights into the mechanism of TRCs induced by Top1ccs, we determined genomic sites of DSBs after Top1cc induction using END-seq technique (*48*). To determine DSBs immediately induced by Top1ccs, we mapped DSBs after 10 and 20 min of CPT treatment since DSBs can already be detected at these short times [see figure 2 in (*18*) and fig. S4, A and B]. We performed two biological replicates in HCT116 cells comparing treated to untreated cells (table S1). After read normalization over library size and spike-in and peak calling for each replicate, we considered strand-specific peaks that were closer than 150 bp to each other as a signal of a DSB (fig. S4C). Then, overlapping DSB sites present in both replicates were merged defining a DSB cluster (fig. S4, D and E; see also Materials and Methods for full description). Since Top1ccs likely revert during the initial cell-handling steps of the END-seq procedure (see Fig. 2B) (*2*, *3*), DSBs are not primarily caused by a Top1cc in one strand and an endonuclease-dependent nick on the other. Read profiles of metaplots of DSB clusters were consistent with END-seq procedures and indicated that a DSB on average was present at the center of the cluster (fig. S4F). However, the clusters were heterogenous including one or a few paired strand-specific peaks (fig. S4G).

**Fig. 4.**
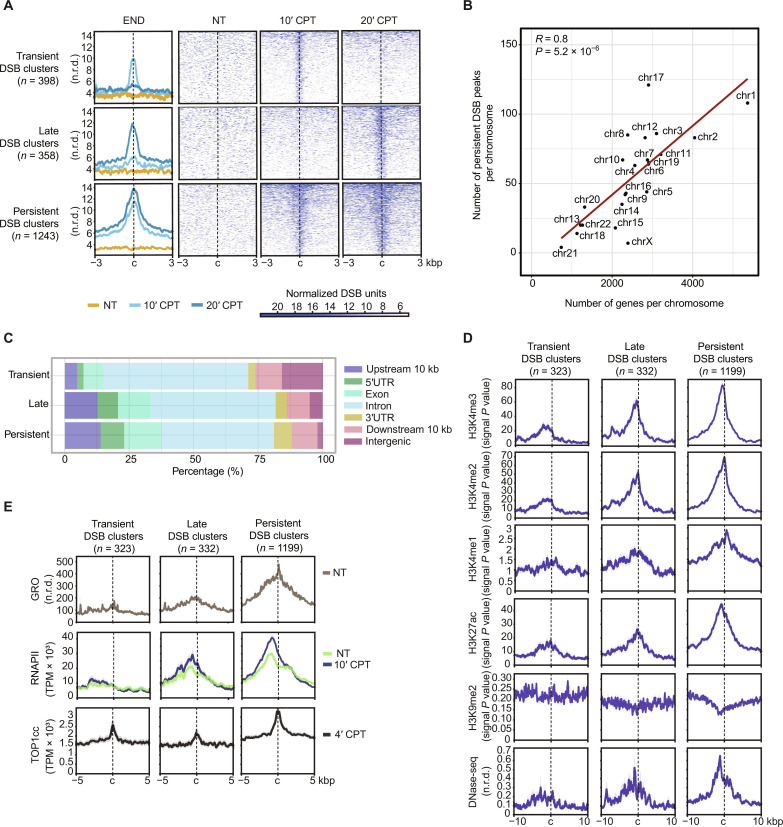
Top1ccs induce DSB clusters after short times in HCT116 cells. (**A**) Metaplot (left) and heat map (right) of END-seq levels normalized read density at transient, late, and persistent DSB regions, showing kinetics of DSBs induced by CPT-mediated Top1ccs at control conditions (NT) and 10 min (10′ CPT) and 20 min (20′ CPT) of treatment. Line colors as in legend. “c” indicates the center of the region in a window of ±3 kbp. All signals are reported as means of two biological replicates normalized levels. (**B**) Scatter plot of correlation between number of genes (*y* axis) and number of persistent DSBs (*x* axis) per chromosome. Spearman correlation coefficient and *P* values are reported. (**C**) Proportion of transient, late, and persistent DSBs over genomic features, as percentage. Colors as in legend. (**D**) Metaplot showing ChIP-seq normalized signals of euchromatin histone markers (H3K4me3, H3K4me2, H3K4me1, and H3K27ac), heterochromatin histone marker H3K9me2, and DNase-seq signals (*39*) at transient, late, and persistent DSB clusters, excluding those localized at intergenic loci. Histone marker ChIP-seq signal is reported as −log_10_(*P* value). DNase-seq signals are reported as normalized read-depth levels. “c” indicates the center of the region in a window of ±10 kbp. (**E**) Metaplot showing GRO-seq (nontreated), RNAPII ChIP-seq (nontreated and 10-min CPT-treated), and Top1cc-seq (4-min CPT) normalized levels at transient, late, and persistent DSB regions, excluding regions localized at intergenic loci. Line colors as in legend. “c” indicates the center of the region in a window of ±5 kbp.

We identified three kinetic groups of DSB clusters: 1243 DSB clusters were persistent, whereas 398 and 358 were transient and late, respectively (Fig. 4A and fig. S4, E and H). In addition, we also detected isolated peaks in one strand only at 10 and 20 min, which are consistent with single-ended DSBs (seDSBs) (fig. S5, A and B). Genomic distribution of persistent DSB clusters correlated well with chromosome gene density (Fig. 4B), consistent with a genic localization of almost all of them (Fig. 4C). However, epigenomic annotations were different among persistent, late, and transient DSB clusters (Fig. 4D). In particular, transient DSB clusters were prevalently found in silent or repressed chromatin, as determined by specific histone marks and deoxyribonuclease (DNase) I hypersensitivity (Fig. 4D). Top1cc levels [mapped previously in HCT116 cells (*6*)] were only slightly higher for persistent DSBs supporting a causative role of them in DSB formation for all the three kinetic groups (Fig. 4E). Persistent DSB clusters were instead associated with higher transcription levels and RNAPII density [mapped previously in HCT116 cells (*6*); Fig. 4E]. Persistent and late DSB clusters were found downstream of RNAPII peaks, whereas they coincided precisely with GRO-seq [mapped previously in HCT116 cells (*38*)] and Top1cc signal peaks (Fig. 4E). As Top1cc induction by CPT causes an increase in RNAPII density at promoter-proximal pause sites (*6*), these findings show that persistent DSBs likely form at specific sites of highly transcribed genes downstream of accumulated RNAPII.

### Top1cc-induced DSBs are enriched at stable, but not transient, hybrid gains downstream of RNAPII peaks

**Fig. 5.**
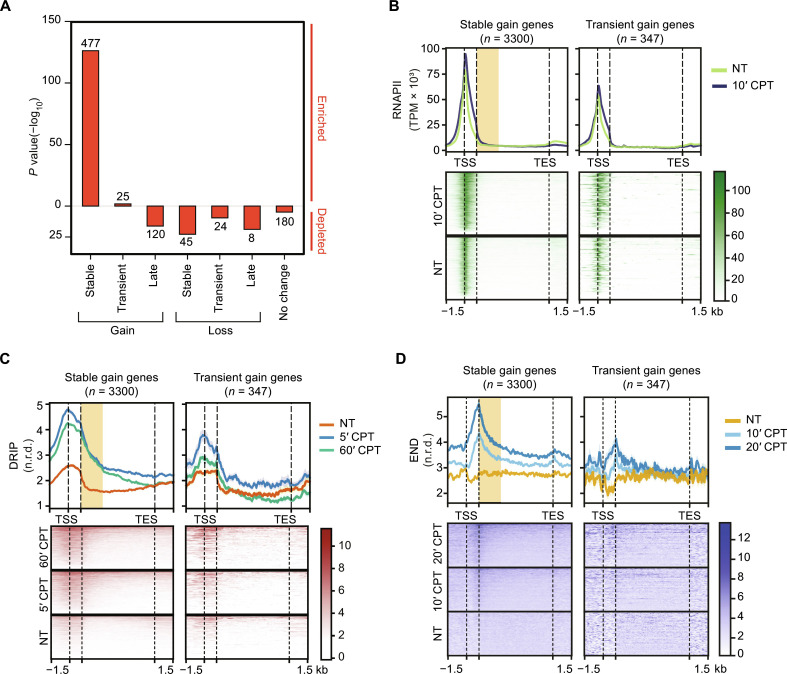
Top1cc-induced DSBs are enriched at stable hybrid gains and are localized immediately downstream of accumulated RNAPII peaks. (**A**) Enrichment analysis of observed persistent DSBs at DRIP categories versus expected by genome randomization (*n* = 100). Bar plots report binomial test *P* values (*y* axis) of enrichment. −Log_10_(*P* values) are referred as “enriched” for a positive overlap enrichment over expected or “depleted” for a depletion over expected. The number of observed overlaps at each category is reported. (**B** to **D**) Metaplots of RNAPII ChIP-seq (B), DRIP-seq (C), and END-seq (D) in genes with annotated stable and transient gain DRIP peaks in a window of ±1.5 kbp. TES, transcription end site. The dashed lines downstream of TSS indicate a 1000-bp unscaled region. The shaded yellow area downstream of the unscaled region indicates a stable gain hybrid region anterior to RNAPII accumulation. All signals are reported as normalized levels. Line colors as in the legend.

Next, to assess whether persistent DSBs were associated with R loops, we computed overlap enrichments and found that DSB clusters were strongly and significantly associated with stable gains only (Fig. 5A, *n* = 477). In addition, seDSB peaks were also enriched with stable gains (fig. S5C). Stable gains overlapping (*n* = 444) with at least one DSB cluster (DSB^+^ stable gains) had only slightly higher hybrid levels than those not overlapping (*n* = 3108) any DSB (DSB^−^ stable gains) (fig. S5D). However, they showed a much higher transcription rate, RNAPII accumulation, and Top1cc signals (fig. S5, E to G). Thus, DSB^+^ stable gains were marked by an elevated transcription suggesting a mechanism linking high transcription to DNA cleavage induced by Top1ccs (*13*).

To determine the relative position of hybrids, DSBs, and RNAPII, we made metaplot analyses aligning the corresponding genes at the TSS and transcription end site (TES) comparing stable to transient hybrid gains. RNAPII peaks were localized close to TSS and overlapping with hybrids for both stable and transient gains (Fig. 5B). Stable gains were downstream of RNAPII, whereas transient hybrid levels were mainly overlapping with and upstream of RNAPII (Fig. 5, B and C, and fig. S6A). RNAPII increase was higher downstream to TSS, suggesting an accumulation of RNAPII induced by Top1ccs (Fig. 5B). Last, DSB tag profiles showed elevated levels in genes with stable gains, showing that DSB clusters, on average, localized remarkably close and downstream of accumulated RNAPII and overlapped with stable hybrid gains (Fig. 5D). Therefore, although our DRIP protocol used enzyme digestion to fragment genomic DNA resulting in an average peak size of 1700 bp (fig. S1E) (*49*), metaplot analyses are consistent with stable hybrid gains being downstream of accumulated RNAPII and often overlapping with DSB clusters.

### Persistent DSB clusters are enriched at early replicating initiation zones and topologically associating domain boundaries

As Top1cc-induced DNA cleavage is known to occur mainly in S phase (*2*, *3*), we next investigated the relationships of persistent DSB clusters with replication origins and timing using short nascent DNA strand (SNS)–seq (*50*) and repli-seq (*51*) annotations, respectively. As high SNS signals [from (*22*)] correspond to replication origin sites (*22*, *50*), persistent DSB clusters did not overlap with replication origins as the SNS signal was depleted (fig. S6B). This is consistent with persistent DSB clusters localized in gene bodies (Fig. 4C) where replication origins are suppressed by transcription (*50*, *52*–*55*). Repli-seq annotations show the timing of replication of large replicating initiation zones (IZs) commonly grouped into early, early/mid, and late IZs (*50*, *51*). The analyses showed that persistent DSB clusters and seDSBs were strongly and highly enriched at early IZs (fig. S6, C and D, respectively), indicating that significant levels of DNA damage triggered by Top1ccs occur during early S phase. We also observed a significant enrichment of all hybrid kinetic categories, but not transient gains, at early IZs (fig. S6E), suggesting that early IZs are enriched with DNA-RNA hybrids.

**Fig. 6.**
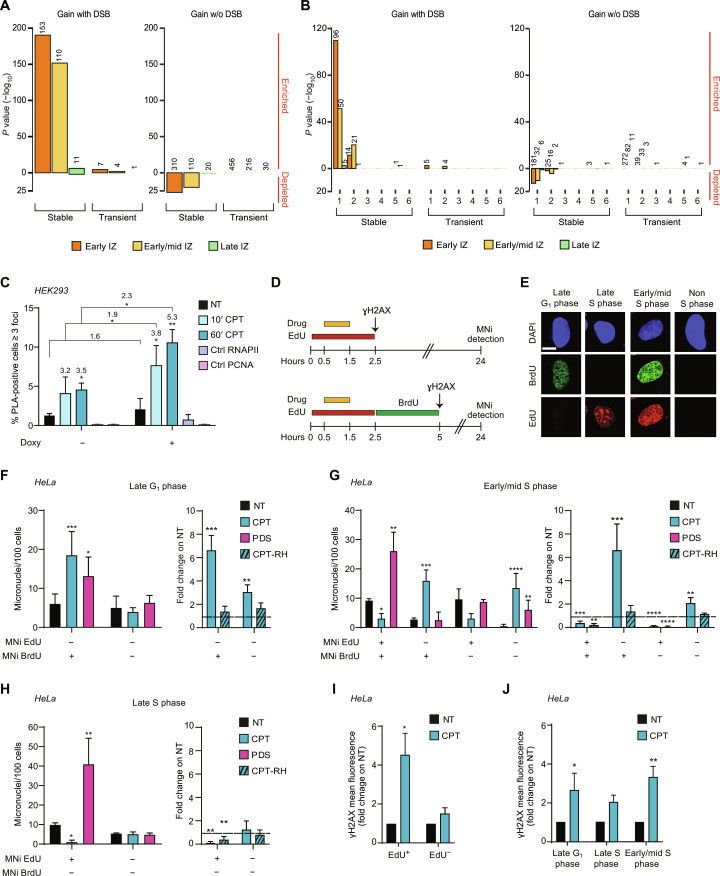
Top1cc-induced DSBs are enriched at early replicating IZs during early/mid S phase causing micronucleus formation. (**A** and **B**) Enrichment analysis of observed R loop gain with and without (w/o) DSBs at early, early/mid, and late IZs (A) and at early, early/mid, and late IZs and in TAD classes (B) assessed as in Fig. 5A. (**C**) PLA assay of PCNA with RNAPII in cells expressing (+ doxy) or not (− doxy) TFIISm. CTRL, control samples using the indicated antibody only. Fold changes of CPT-treated versus untreated and induced versus uninduced samples are reported over each bar. Mean of analyzed cells is 800. Statistical significance was calculated with one-tailed paired *t* test. (**D**) Experimental protocol of 1-hour CPT treatment with single (top) or dual (bottom) labeling of HeLa cells. (**E**) Representative images of the possible cases of dual labeled cells. (**F** to **H**) Levels of micronuclei triggered in late G_1_, late S, or early/mid S phase [as in (E)], respectively. Micronucleus levels are reported as micronuclei (MNi) per 100 cells (left) or as fold change in RNase H1–expressing (RH) versus control cells (right). Means of analyzed cells are 250 (F), 150 (G), and 400 (H). Statistical significance was calculated comparing micronucleus distribution of treated over untreated samples with two-tailed Mann-Whitney test. (**I** and **J**) γH2AX detected by immunofluorescence microscopy at the end of EdU administration (I) or at the end of BrdU administration (J) as in (D). Levels of nuclear fluorescence are reported as fold change over untreated cells (number of analyzed cells is 450). Statistical significance was calculated considering treated over untreated samples with one-tailed ratio paired *t* test. In (C) and (F) to (J), bars show means ± SEM of three biological replicates. *P < 0.05; **P < 0.01; ***P < 0.001; ****P < 0.0001.

Next, we wondered whether stable hybrid gains with persistent DSB clusters had a preferential genomic location. The results show that DSB^+^ stable gains (263) were strongly enriched at early or mid IZs (*51*), whereas stable gains without a DSB cluster were depleted (Fig. 6A). Since topologically associating domain (TAD) boundaries can overlap early replication regions (*51*, *56*), we also measured the enrichments of DSBs and hybrid gains at these chromatin elements. Class 1 TAD boundaries, which have been shown to be enriched at early replicating IZs (*51*), showed a significant enrichment of R loops, which are not enriched at other TAD boundary classes (fig. S6F). Moreover, Top1cc induction overall decreased hybrid levels at class 1 TADs (fig. S7, A and B). Enrichment analyses showed that DSB^+^ stable gains (146), but not DSB-stable gains, were clearly associated with early or early/mid IZs of class 1 TAD boundaries (*51*) (Fig. 6B). In contrast, transient gains with or without DSBs were not enriched in any IZ or TAD class (Fig. 6, A and B). Metagene analyses of DSB^+/−^ stable gains overlapping early versus early/mid IZs found in class 1 TAD boundaries showed that hybrid levels were increased by Top1ccs, and their levels were higher in early versus early/mid IZs (fig. S7C). RNAPII density and DSB levels also increased in Top1cc induction and both peaked close to gene 5′-ends (fig. S7C). Thus, the results support that Top1cc-induced replication fork collisions occur with stable hybrid/RNAPII leading to DSB clusters at early/mid replicating IZs.

Next, we asked the question of whether the elongation factor TFIIS has any role in TRCs induced by Top1ccs. We thus performed PLA assays using antibodies against proliferating cell nuclear antigen (PCNA) (PC11, sc-53407) and the largest subunit of RNAPII (H-224, sc-9001) to determine the proximity of replication forks to RNAPII upon Top1cc induction (fig. S7D). The results clearly showed that TRCs are markedly increased by TFIISm (Fig. 6C) and are dependent on replication and transcription as specific inhibitors markedly reduced PLA signals (fig. S7E). The findings thus support the role of WT TFIIS in attenuating Top1cc-induced TRCs by releasing arrested RNAPII.

### Micronuclei are triggered by Top1cc induction at specific cell cycle phases and enhanced by TFIISm

Since Top1cc-induced DSBs are enriched at early and early/mid replicating IZs (Fig. 6, A and B), we therefore wonder whether Top1cc triggering of micronuclei, a marker of genome instability (*4*, *18*), occurs preferentially during early S phase. Therefore, we induced Top1ccs for a brief time (1 hour) and then counted micronuclei after 24 hours of cell growth in drug-free medium. During Top1cc induction, S phase cells were labeled with 5-ethynyl-2′-deoxyuridine (EdU) to distinguish, at the time of micronucleus counts, cells that were in S phase (EdU^+^) from those that were not (EdU^−^) during Top1cc induction (experimental scheme in Fig. 6D, top, and fig. S8A). The results showed that micronuclei were increased in EdU^+^ and EdU^−^ cells, showing that Top1ccs can trigger the formation of micronuclei during S and other phases (fig. S8B). In particular, Top1ccs induced micronuclei with EdU^−^ staining, suggesting that micronucleus DNA was not replicated during CPT treatment. RNase H1 expression (fig. S8, C to F) reduced Top1cc-induced micronuclei in both EdU^+^ and EdU^−^ cells (fig. S8C). The reduction was not complete because of cell heterogeneity of RNase H1 expression (fig. S8, D and E), as determined by dose/response effects previously (*18*). Breast cancer type 2 susceptibility protein (BRCA2) gene silencing increased Top1cc-induced micronuclei (fig. S8G), and its effect was fully abolished by meiotic recombination 11 homolog A (MRE11) inhibition (fig. S8, H and I) in EdU^−^ cells, showing that impairment of fork stabilization and/or cleavage processing and repair can mediate micronucleus formation upon Top1cc induction in non–S phase cells. As micronuclei can be a consequence of mitotic errors, we also determined anaphase bridges (marker of abnormal mitoses) induced by Top1ccs (fig. S8J). We found that anaphase bridges and ultrafine bridges were induced by Top1ccs in EdU^+^ and EdU^−^ cells and, in part, suppressed by RNase H1 expression (fig. S8, K and L). Thus, genome instability by Top1ccs originated at both S phase and non–S phases in a hybrid-dependent way.

Next, to better define cell cycle phase specificity, we performed a dual-labeling protocol by adding a second label [5-bromo-2′-deoxyuridine (BrdU)] of DNA replication immediately after EdU removal (Fig. 6D, bottom). We thus defined four classes of cells based on the phase at the time of Top1cc induction: early/mid S (EdU^+^/BrdU^+^), late S (EdU^+^/BrdU^−^), late G_1_ (EdU^−^/BrdU^+^), and non-S (EdU^−^/BrdU^−^) (Fig. 6E). The findings demonstrated that Top1ccs trigger micronucleus formation via a hybrid-mediated mechanism specifically at late G_1_ and early/mid S phases, but not at late S phase (Fig. 6, F to H, and fig. S8M). Pyridostatin [a G-quadruplex ligand (*57*, *58*)] induced micronuclei in all phases (Fig. 6, F to H), showing that replication stress during late S may indeed lead to micronucleus formation. As expected, Top1ccs increased Ser139-phopshorylated H2AX histone (γH2AX) foci in S phases, but not in non–S phases (Fig. 6I); however, γH2AX foci formed upon transition from G_1_ to S phase in cells exposed to CPT during late G_1_ phase (Fig. 6J and fig. S8N), consistent with replication fork collisions with stable hybrid/RNAPII at early replicating IZs.

**Fig. 7.**
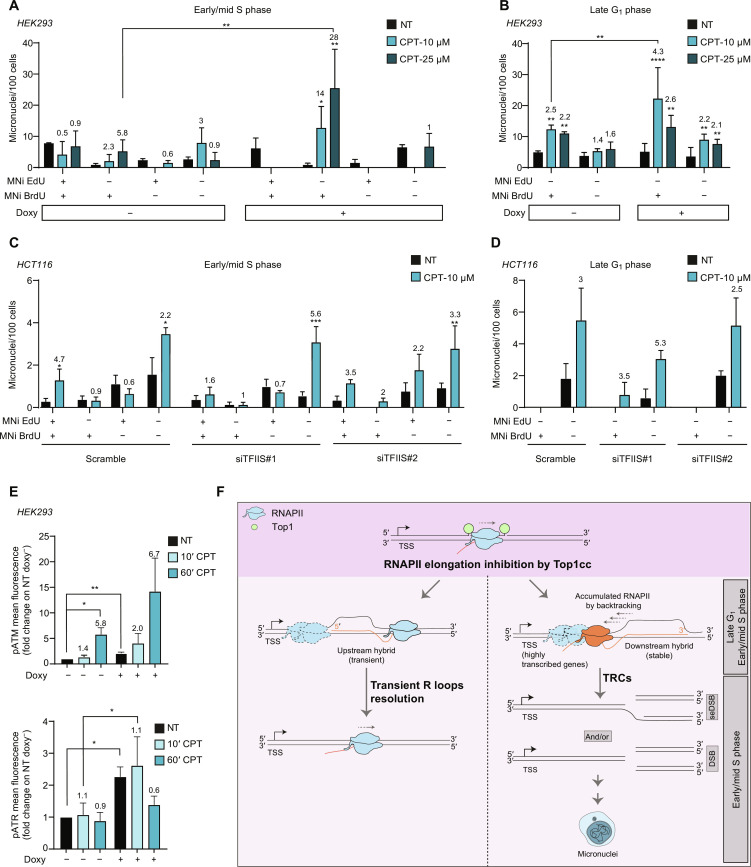
Effects of elongation factor TFIISm on micronucleus formation. (**A** and **B**) Micronucleus quantitation in HEK293 cells overexpressing or not TFIISm that were in the indicated cell cycle phase during 1-hour CPT treatment. Micronuclei were distinguished on the basis of EdU and BrdU incorporation (EdU^+/−^ and BrdU^+/−^). Micronucleus levels are reported as micronuclei per 100 cells (mean of analyzed cells is 200). Each bar represents the mean values ± SEM of three biological replicates. Statistical significance was calculated comparing micronucleus distribution of treated over nontreated samples with two-tailed Mann-Whitney test. (**C** and **D**) Micronucleus quantitation in HCT116 cells silenced for TFIIS, with two different small interfering RNA (siRNAs) (siTFIIS#1 and siTFIIS#2), that were in early/mid S phase (C) or late G_1_ phase (D) during 1-hour CPT treatment. Micronucleus levels are reported as micronuclei per 100 cells. Mean of analyzed cells is 200 (D) and 50 (E). Each bar is the mean values ± SEM of three biological replicates. Statistical significance was calculated comparing micronucleus distribution of treated over nontreated samples with two-tailed Mann-Whitney test. (**E**) ATM S1981 and ATR S428 phosphorylations detected by immunofluorescence in HEK293 cells induced (doxy^+^) or not (doxy^−^) for TFIISm expression. Levels of nuclear fluorescence are normalized on uninduced control sample (NT). Fold change of CPT treated versus untreated samples is reported over each bar (mean of analyzed cells is 400). Each bar represents the mean values ± SEM of three biological replicates. Statistical significance was calculated considering data normalized on NT sample with one-tailed ratio paired *t* test. In all panels, *P < 0.05; **P < 0.01; ***P < 0.001; ****P < 0.0001. (**F**) Model of R loop increase and TRCs induced by Top1ccs. The model illustrates head-on TRCs only as codirectional TRCs are not shown for simplicity.

We then investigated the role of elongation factor TFIIS on micronucleus formation (fig. S9A). The expression of TFIISm (Fig. 3B) (*46*) markedly increased Top1cc-induced micronuclei when cells were exposed to CPT during early/mid S and late G_1_ phases, but not in late S phase, even at a higher CPT concentration (Fig. 7, A to B, and fig. S9, B and C). Consistent with previous observations (Fig. 6G and fig. S8B), Top1ccs induced BrdU^+^ but not EdU^+^ micronuclei in early/mid S phase category (Fig. 7A), consistently with synthesis-dependent repair of DSBs, leading to micronuclei, often occurring later than Top1cc induction and DSB formation. Similarly, TFIIS silencing (fig. S9D) increased Top1cc-induced micronucleus levels specifically in early/mid and late G_1_ phases (Fig. 7, C and D, and fig. S9, E and F). Moreover, we measured Ataxia Telangiectasia Mutated protein (ATM) and Ataxia Telangiectasia and Rad3-related protein (ATR) activation upon Top1cc induction at single-cell levels. Top1ccs triggered phosphorylation of ATM S1981 markedly, whereas ATR phosphorylation of ATR S428 was barely or not increased (Fig. 7E and fig. S9G). TFIISm expression increased both ATM and ATR phosphorylations in control cells; however, it increased even further Top1cc-induced ATM, but not ATR, phosphorylation (Fig. 7E). Together, the findings show that TRCs leading to DSBs and micronuclei occur at early/mid replicating IZs and the elongation factor TFIIS can attenuate the genome instability effects by reducing TRC formation (Fig. 7F).

## DISCUSSION

We provide evidence of a main mechanism of Top1cc-induced TRCs and genome instability in human cancer cells. As Top1ccs are known to induce DNA cleavage in a manner dependent on R loops and replication, we have determined TRC sites by genome maps of hybrids and DSBs rapidly following Top1cc induction. Our results define the genomic sites of Top1cc-associated DSBs, which are likely due to DNA-RNA hybrids downstream of arrested RNAPII and TRCs resulting in fork collapse, DNA cleavage, and micronucleus formation. Top1cc-triggered mechanisms are rapid dynamic processes, consistently with Top1ccs being reversible even in the presence of CPT (*3*, *59*). Moreover, Top1ccs are close to hybrid gains, and, because of that, Top1cc-related strand breaks destabilize hybrids during DRIP protocols. Thus, the presence of nearby strand cuts can affect hybrid detection reducing the efficiency of mapping protocols, particularly upon Top1cc induction.

Regions of Top1cc-induced R loop changes are different from sites of R loop alterations by Top1 gene silencing (*60*). Top1ccs induce hybrid gains mainly at gene regions through distinct mechanisms (Fig. 7F). Hybrid gains at 5 min of CPT were either persistent (stable gains) or transient (transient gains), depending on whether they were observed at 60 min, respectively. The two classes differ in many aspects such as peak length, specific chromatin features, and distance from the gene 5′ end. In contrast to transient gains, stable gains map downstream of RNAPII and are strongly associated with DSB clusters induced by Top1ccs. These DSB clusters are strongly enriched in early replication IZs and TAD boundaries. Considering that Top1ccs also increased genomic hybrids after 60 but not 5 min of CPT treatment (late gains), the results show that Top1ccs induce diverse types of R loops likely through different mechanisms. Transient hybrid gains, which are largely upstream of RNAPII, are likely due to increased negative torsional tension behind RNAPII and/or to the presence of a DNA nick that could allow rotation of DNA/RNA strands around each other to form the heteroduplex (*32*). On the other hand, stable hybrids, which are downstream of RNAPII, can be increased following RNAPII backtracking (Fig. 7F) (*46*). Late hybrid gains are likely due to yet undefined molecular events following the overall cellular stress caused by Top1ccs. Overall, our findings show that R loops can form with different mechanisms although they have a common trigger, Top1ccs, showing differences in persistence and structural aspects in living cells. Therefore, other conditions known to increase nuclear R loops, but for which no evidence of RNAPII backtracking has been implied, may lead to R loops with structural differences affecting their resolution, genome stability, cell cycle phase enrichment, gene expression, and epigenetic regulation.

We have here focused on stable hybrid gains and persistent DSBs as we found highly significant enrichments of DSB clusters at highly transcribed sites associated with stable hybrids downstream of accumulated RNAPII at early replicating IZs (Figs. 4 and 5). Genome instability effects of a dominant-negative mutation of TFIIS are consistent with a mechanism of TRC-mediated, at least in part, by RNAPII backtracking (*46*). Top1 interacts with and is activated by RNAPII to allow efficient elongation by coupling RNAPII activity to the removal of duplex torsional tension (*6*, *44*). Thus, forming along highly transcribed genes, CPT-stabilized Top1ccs can impede RNAPII elongation increasing the probability of RNAPII backtracking, which may even reach regions close to promoter-proximal pause sites, allowing the formation of stable downstream R loops (Fig. 7F). A backtracked, arrested RNAPII associated with a stable R loop can be a significant block for an advancing replication fork (*61*), particularly at highly transcribed genes where a backtracked RNAPII can cause the arrest of many other upstream RNAPIIs (Fig. 7F). Bacterial RNA polymerase backtracking has been shown to cause TRCs in prokaryotes by increasing hybrids and DSB levels, suggesting that the mechanism may be evolutionary conserved (*62*–*64*). As we found strong enrichments of DSBs and stably increased hybrids at early replicating IZs, the DSB clusters likely correspond to main sites of TRCs in human HCT116 cancer cells. This mechanism is supported by functional results as Top1cc induction at G_1_/early-mid S phases, but not at late S phases, can trigger BRCA2/Mre11- and hybrid-dependent micronuclei, which are attenuated by TFIIS.

The dominant-negative TFIIS variant induced RNAPII accumulation mainly at the 5′-end of expressed genes with a downstream shoulder [see figure 2D in (*46*)] remarkably similar to RNAPII accumulation upon Top1cc induction (Figs. 3 and 5) [see also (*6*)]. This indicates that backtracked RNAPII sites are frequent at gene 5′-ends and the polymerase becomes arrested close to proximal-promoter pause sites (*46*). In addition to the 5′-end, gene 3′-ends are also a main region of RNAPII and TFIIS accumulation (*47*), where genomic data support that R loops can, in part, be downstream of RNAPII peaks (Fig. 3). Consistently, although DSB clusters are prevalently located close to 5′-ends of genes, we found DSBs along the full gene length, particularly at transcription termination regions (Fig. 5).

Top1cc-induced DSBs are rapid and mapped at selective genomic sites. A high fraction of persistent DSBs (21%) overlapped with early and early/mid replicating IZs (*51*), and most (56%) of which were mapped at class 1 TAD boundaries (*51*). As DSBs were mapped in an asynchronous cell population, a significant level of TRCs leading to DSBs occurs at early replicating IZs during G_1_/early-mid S phases. Top1ccs have been mapped at nucleotide levels at replication origin of Lamin B2 gene at late G_1_ and G_1_-S transition in human cancer cells (*65*). In this study, the authors detected Top1ccs in G_1_, before the start of DNA synthesis, leading to firing inhibition in S phase (*65*). As Top1ccs can induce firing of dormant origins (*66*, *67*), whether TRCs originate from excess activation of replication origins early in S phase remains to be determined.

The rapid formation of DSBs raises the question of which are the processes that rapidly respond at TRC sites making DNA strand cuts. Although CPT was expected to induce specifically seDSBs (*3*, *19*, *20*), our data show that TRCs rapidly generate DSBs with at least two ends, as detected by END-seq technique (Fig. 4), along with seDSBs (fig. S5, A and B), which could also be a sign of fork reversal. One possibility is that seDSB cuts are generated by DNA polymerase runoff at Top1cc sites of template strands (*3*, *19*, *20*). Next, seDSBs may be converted to triple-end DSBs at collapsed forks in which the second template is cleaved by specific enzymes (Fig. 7F). Alternatively, replicative nucleases may directly cause the breakage of both templates. The structure specific Methyl methansulfonate, UV sensitive 81 (Mus81)/Essential Meiotic Endonuclease 1 (Eme1) endonuclease has been proposed to protect stalled forks and to restart replication (*21*–*25*), and it could contribute significantly to DSB clusters formed after a brief time from Top1cc induction. Other endonucleases have also been proposed to repair Top1cc-mediated DNA cleavage (*4*, *68*). However, specific enzyme roles in generating the observed DSB clusters need to be addressed in the future.

A limitation of the study is the low resolution of DRIP-seq protocol, which uses restriction enzymes to fragment genomic DNA (*49*). In this study, hybrid peak size is around 987 bp for unchanged peaks, whereas it is 2618 and 812 bp for stable and transient gains, respectively (fig. S1E). Thus, although our analyses show a clear different location of stable and transient hybrid gains relative to RNAPII (downstream and upstream, respectively), the low resolution impedes a precise mapping of the hybrid at each individual site. Similarly, although we show a substantial overlap of stable gains with DSBs, we cannot define their location at higher resolution at each individual gene. Thus, the location of stable hybrids in relation to RNAPII remains to be defined at nucleotide levels to understand further aspects of RNAPII arrest and downstream hybrid formation.

In summary, our findings reveal an unexpected mechanism of TRCs and genome instability elicited by unbalanced Top1ccs, establishing a strong role of high transcription rate at early replicating IZs as a source of genome instability. In particular, the findings document a significant role for arrested RNAPII (Fig. 7F) and can open to the development of alternative strategies to prevent and cure neurodegenerative and cancer diseases.

## MATERIALS AND METHODS

### Cell lines and treatment

Human cancer HCT116 cells, U2OS, and HeLa cell lines were purchased from American Type Culture Collection (LGC Standards S.r.l., Milan, Italy). HCT116 cells were cultured in a humidified incubator at 5% of CO_2_ and 37°C in McCoy’s 5A medium supplemented with 10% heat-inactivated fetal bovine serum (FBS). U2OS and HeLa cell lines were cultured in Dulbecco’s modified Eagle’s medium (DMEM) supplemented with 10% heat-inactivated FBS. U2OS-RH cells have been previously generated by transfecting U2OS with pLVX-Tight-Puro-RH-Flag and pLVX-EF1a-Tet3G-Hygro plasmids to overexpress an exogenous RNase H1 (*69*). This cell line was maintained by adding 10% FBS tetracycline-free (Takara, #631106), penicillin-streptomycin (100 μg/ml), puromycin (1.5 μg/ml), and hygromycin B (500 μg/ml) to DMEM medium. RNase H1 overexpression was induced, 24 hours after seeding, by adding doxycycline (2 μg/ml) for 48 hours to the same medium without hygromycin and puromycin. To overexpress RNase H1 in HeLa cells, after 24 hours from seeding, cells were transiently transfected with 2.5 μg of pcDNA3–RNase H1 (gently furnished by F. Chedin) using Lipofectamine 2000 (Thermo Fisher Scientific, #11668027) with a ratio plasmid (in micrograms):Lipofectamine 2000 (in microliters) of 1:2. pcDNA3–RNase H1 plasmid was purified by using miniprep kit (Macherey-Nagel, #740588.50) from *Escherichia coli* DH5α cells grown in LB medium with ampicillin (100 μg/μl). HeLa and HCT116 cells were transfected with predesigned small interfering RNA (siRNAs) against BRCA2 (QIAGEN, ID #sI02653595, siRNA) (*69*) and TFIIS (Ambion–Thermo Fisher Scientific; ID# s13847, siRNA1; ID# s13848, siRNA2) respectively, using Lipofectamine RNAiMAX (Invitrogen) for 24 hours following the manufacturer’s instructions. HEK293-TFIISm cells stably express a doxycycline-inducible full-length mutant of TFIIS Transcription Elongation Factor A1 (TCEA1) (*46*). This cell line was kindly supplied by J. Q. Svejstrup (CGEN, University of Copenhagen, Denmark) and was cultured in DMEM supplemented with 10% FBS tetracycline-free, penicillin-streptomycin (100 μg/ml), zeocin (250 μg/ml), and blasticidin (5 μg/ml). TFIISm overexpression was induced by adding doxycycline (80 ng/ml) for 48 hours to the same medium without antibiotics. END-seq spike-in cell line was gently furnished by A. Nussenzweig (National Institutes of Health). Murine pre–B cells [Lig4^−/−^, zinc-finger endonuclease (ZFN) endonuclease inducible] were cultured in DMEM complemented with 10% FBS, 2 mM l-glutamine, penicillin-streptomycin (100 μg/ml), 1 mM sodium pyruvate, 1× nonessential amino acids, and 55 μM β-mercaptoethanol.

### Establishment of HeLa stable cell line expressing the catalytically modified RNase H1

HeLa cells at 90% confluency were transfected using Lipofectamine 2000 (Thermo Fisher Scientific, #11668019) with the pPyCAG–RNase H1 WT, pPyCAG–RNase H1 D210N, or pPyCAG–RNase H1 W43A, K59A, K60A, D210N (WKKD) (Addgene, #111904, #111905, and #111906), following the manufacturer’s instructions. For each 10-cm cell dish, 24 μg of plasmid has been added to 60 μl of Lipofectamine 2000 in Opti-MEM (Thermo Fisher Scientific, #31985062), and the solution was added to growing cells. After 24 hours, the cell medium was replaced with fresh DMEM (Thermo Fisher Scientific, #21969035), and 2 days after transfection, hygromycin B (Thermo Fisher Scientific, #10687010) was added to a final concentration of 300 μg/ml. This selection process lasted about 4 weeks, and then cells were checked for expression level by Western blot.

### DRIP method and DRIP-seq

DRIP assay was performed as in (*70*) with minor modifications as hereafter reported. A total of 3 × 10^6^ HeLa, 3.5 × 10^6^ HCT116 cells, and 3.5 × 10^6^ TFIISm-expressing HEK293 were seeded in 100-mm dishes and treated, after 24 hours, with 10 μM CPT for 5 and 60 min. After treatment, non–cross-linked cells were directly lysed or washed with 1× phosphate-buffered saline (PBS) and recovered in drug-free medium for 15 min. Cell scrapers were used to harvest cells in lysis buffer [10 mM tris-HCl (pH 7.6), 1 mM EDTA (pH 8.0), 0.5% SDS, and proteinase K (62.5 μg/ml)]. Genomic DNA was enzymatically digested with 200 U of Hind III [New England Biolabs (NEB), #R0104S], Eco RI (NEB, #R0101S), Xba I (NEB, #R0145S), Ssp I (NEB, #R0132L), and Bsr GI (NEB, #R0575L) restriction enzymes in buffer 2.1 (NEB, #B7202S) with the addition of bovine serum albumin (BSA) (0.2 mg/ml; NEB, #B9000S). After digestion, half of genomic DNA was treated overnight (O/N) at 37°C with RNase H (NEB, #M0297L). At this point, 5 μg of genomic DNA (treated or not with RNase H) was immunoprecipitated for 2 hours at 4°C with 40 μl of protein A magnetic beads (Thermo Fisher Scientific, #10002D) previously incubated O/N at 4°C with 6 μg of S9.6 antibody (homemade). On the other side, 1 μg of digested DNA (treated and untreated with RNase H) was used as input. Enrichment over input (percentage of input) per sample was quantified by real-time PCR. For DRIP-seq, two biological replicates were performed, and each replicate consisted of two DRIP samples conducted in parallel and then pooled before the sonication step. Treated and untreated cells were harvested by using Accutase (Thermo Fisher Scientific, #A11105-01) and then washed with PBS and pelleted for a total of 15 min. Then, cells were lastly lysed as reported in (*70*). Therefore, cells were lysed after 15 min from the end of CPT treatment. To increase the efficiency of library preparation, all samples were treated for 1 hour with 1 μl of RNase H at 37°C and then purified with StrataClean Resin (Agilent, #400714) and Sephadex G-50 column (Merck, #G5080). To concentrate the samples, genomic DNA was ethanol-precipitated and lastly resuspended in 50 μl of RNase/DNase-free water. Samples were then sonicated with Bioruptor Sonicator (Diagenode) for 20 min in pulse/pause mode setting (15-s on/30-s off; high intensity; 4 cycles of 5 min each). Library preparation and sample sequencing by Illumina paired-end (75 + 75–bp reads) sequencing technology was carried out by the genomic unit of CABIMER (Centro Andaluz de Biología Molecular y Medicina Regenerativa) (Seville, Spain). In DRIP-seq and DRIP-qPCR experiments, an internal standard (spike-in) was used to reduce technical variability among the three biological replicates.

### DRIP(-seq) spike-in

To map and measure R loop levels, two diverse types of spike-in were prepared. To perform DRIP-seq experiments, genomic DNA from *Saccharomyces cerevisiae* was used. DRIP protocol described in (*70*) was used to extract yeast genome. Briefly, yeast mid-log cultures growing in standard yeast extract peptone glucose adenide sulfate (YPAD) medium at 30°C were collected, resuspended in 1.4 ml of spheroplasting buffer [1 M sorbitol, 10 mM EDTA (pH 8), 0.1% β-mercaptoethanol, and Zymolyase 20T (2 mg/ml)], and incubated at 30°C for 30 min. At this point, after breaking spheroplasts with 1.65 ml of buffer G2 [800 mM guanidine HCl, 30 mM tris-Cl (pH 8), 30 mM EDTA (pH 8), 5% Tween 20, and 0.5% Triton X-100], purified genomic DNA was digested O/N with restriction enzyme cocktails (50 U of Hind III, Eco RI, Xba I, Ssp I, and Bsr GI). After phenol/chloroform purification, genomic DNA was aliquoted and stored at −80°C to avoid multiple freeze-thaw cycles. Immediately before the immunoprecipitation step, yeast genome was added to the samples with a yeast:human genome ratio of 1:1000. Yeast Pyruvate decarboxylase (PDC1) locus was used to verify the recovered percentage of input and to normalize DRIP results.

For DRIP-qPCR experiments, pFC53 plasmid was used as spike-in in addition to yeast genome. pFC53 plasmid (kind gift from F. Chedin), which contains the mouse Airn (mAirn) CpG island controlled by the T3 promoter, was purified by using miniprep kit (Macherey-Nagel, #740588.50) from *E. coli* DH5α cells grown in LB medium with ampicillin (50 μg/μl). In vitro transcription was performed as hereafter reported. T3 RNA polymerase (4.5 μl; Promega, #P2083) was added to 45.5 μl of a previously prepared mix containing 3 μg of pFC53 plasmid, 1× transcription optimized buffer, 20 mM dithiothreitol, 0.05% Tween 20, 50 μM triphosphate ribonucleotides (rNTPs) (Promega, #P1132, #P1142, #P1152, and #P1162). Transcription was carried out for 30 min at 37°C, followed by enzyme inactivation at 65°C for 10 min. At this point, half of transcribed plasmid (sample A) was treated with 5 μl of RNase A (0.1 mg/ml; Merck, #R6513), while the other half (sample B) with 5 μl of RNase A (0.1 mg/ml) and 2 μl of RNase H (NEB, #M0297L) at 37°C for 30 min. To remove the enzymes, proteinase K (Thermo Fisher Scientific, #EO0491) was added at a final concentration of 1 mg/ml for other 30 min at 37°C, and samples were purified by phenol/chloroform procedure. After confirming R loop formation by running the in vitro transcription product on a 0.8% agarose gel, sample B was further digested and linearized by using ApaLI restriction enzyme (Thermo Fisher Scientific, #AR0041). Digested and purified plasmid from sample B was lastly added to DRIP samples immediately before the immunoprecipitation step with a plasmid:human genome ratio of 1:6 × 10^4^. “R loop fragment” (RF) pair of primers was used to verify the recovered percentage of input and to normalize DRIP results.

### ChIP assay

ChIP experiments have been performed as previously published in (*8*). Briefly, to perform RNAPII and TFIIS ChIP, HEK293-TFIISm cells, induced or not induced with doxycycline, were treated or not for 10 min with 10 μM CPT (Merck, #C9911). Cells were cross-linked with 1% formaldehyde (Merck, #47608) for 10 min at room temperature (RT) and then incubated for further 10 min with 125 mM with glycine (Merck, #G8898). Cross-linked cells were lysed by resuspending and incubating cells in cell lysis buffer [5 mM Pipes (pH 8), 85 mM KCl, 0.5% NP-40, and 1× protease inhibitor cocktail (Thermo Fisher Scientific, #78439)] for 10 min in ice. Samples were then pelleted and resuspended in radioimmunoprecipitation assay (RIPA)–sonic buffer [50 mM tris-HCl (pH 8), 150 mM NaCl, 1% NP-40, 0.5% SDS, and 1× protease inhibitor cocktail] for 20 min in ice. For each sample, chromatin from 10^7^ cells was fragmented with Bioruptor Sonicator (Diagenode) by sonication for 15 cycles in pulse/pause mode setting (30-s on/45-s off; low intensity). Further, 60 cycles of sonication at high intensity were performed picketing samples every 15 cycles. Sonicated chromatin was centrifuged at 20,000*g* for 15 min at 4°C, and the supernatant was precleared for 30 min with 20 μl of BSA-coated beads on a rotating plate. To prepare protein A/G Magnetic Beads (Thermo Fisher Scientific, #88803), 60 μl of beads per sample were previously incubated for 6 hours with 20 μg of BSA (NEB, B9000S). Five percent of precleared chromatin was saved as input, while the resting chromatin was diluted with RIPA–sonic buffer without SDS (to a final concentration of SDS, 0.18%) and incubated O/N at 4°C on a rotating plate with 5 μg of RNAPII (Santa Cruz Biotechnology, sc-47701) or TFIIS (Abcam, ab185947) antibodies. Nonspecific immunoglobulin G (Merck, #M7023; Cedarlane, #CLRB00) was used as negative controls. The next day, 30 μl of BSA-coated beads were added for an additional hour under rotation at RT. Beads were then washed five times with RIPA wash buffer [50 mM tris-HCl (pH 8), 150 mM NaCl, 1% NP-40, 0.1% SDS, and 1× protease inhibitor cocktail], once with LiCl wash buffer [100 mM tris-HCl (pH 8), 1% NP-40, 500 mM LiCl, and 1× protease inhibitor cocktail] and twice with TE buffer [10 mM tris-HCl (pH 8) and 1 mM EDTA]. Washed beads were resuspended in TE buffer, treated with RNase A (Thermo Fisher Scientific, #EN0531), and then decross-linked with proteinase K (Thermo Fisher Scientific, #EO0491) O/N at 65°C. RNase A treatment and reverse cross-link were also performed for input samples. The DNA from all the samples was subsequently purified with phenol/chloroform extraction, followed by ethanol precipitation and pellets resuspended in 50 μl of 1× TE buffer. Specific DNA fragments recovery was determined by real-time PCR.

### R-ChIP method

R-ChIP procedure has been strictly performed as previously published (*43*). Briefly, 24 hours after seeding, HeLa cells stably expressing RNase H1 WT, D210N, or WKKD were treated or not for indicated times with 10 μM CPT (Merck, #C9911). Cells were then cross-linked with 1% formaldehyde (Merck, #47608) for 15 min at RT, and fixation has been stopped by addition of 125 mM glycine (Merck, #G8898) for 15 min at RT. Cells were washed with PBS and scraped off from the plate, and nuclei were extracted with cell lysis buffer [10 mM tris-HCl (pH 8.0), 10 mM NaCl, 0.5% NP-40, and 1× protease inhibitor cocktail] and then suspended in nuclei lysis buffer [50 mM tris-HCl (pH 8.0), 10 mM EDTA, 1% SDS, and 1× protease inhibitor cocktail]. Chromatin DNA was sheared to 200 to 500 bp in size by sonication with Bioruptor Sonicator (Diagenode). Five percent chromatin fragment was saved as input, and the remaining was incubated with magnetic beads conjugated with anti-V5 antibody (Abcam, #ab15828) O/N at 4°C. Beads were sequentially washed three times with wash buffer I [20 mM tris-HCl (pH 8.0), 150 mM NaCl, 1% Triton X-100, 0.1% SDS, 2 mM EDTA, and 1× protease inhibitor cocktail], three times with wash buffer II [20 mM tris-HCl (pH 8.0), 500 mM NaCl, 1% Triton X-100, 0.1% SDS, 2 mM EDTA, and 1× protease inhibitor cocktail], once with wash buffer III [10 mM tris-HCl (pH 8.0), 250 mM LiCl, 1% NP-40, 1% feoxycholate, 1 mM EDTA, and 1× protease inhibitor cocktail], and once with TE buffer [10 mM tris-HCl (pH 8.0) and 1 mM EDTA]. The protein-chromatin complex was eluted with elution buffer [10 mM tris-HCl (pH 8.0), 1% SDS, and 1 mM EDTA] and decross-linked by incubation O/N at 65°C. After sequential RNase A and proteinase K treatment (Thermo Fisher Scientific, #EN0531 and #EO0491), the precipitated hybrid fragment was cleaned by phenol twice and phenol:chloroform:isoamyl alcohol once (Merck, #77617), followed by ethanol precipitation. The recovered fragment was subjected to qPCR.

### K^+^/SDS precipitation

To detect Top1cc, K^+^/SDS precipitation method was used as reported in (*41*) with minor modifications as here reported. Twenty-four hours after seeding, 1 × 10^7^ cells were treated with 10 μM CPT for 5 min. After treatment, cells were directly lysed with lysis buffer [1% SDS, 10 mM tris-HCl (pH 8), 10 mM EDTA, and 1× protease inhibitor) or washed with 1× PBS and recovered in drug-free medium for 15 min. Lysed cells were then sonicated with Bioruptor Sonicator (Diagenode) for 15 min in pulse/pause mode setting (30-s on/30-s off; medium intensity). Chromatin was then quantified at spectrophotometer and diluted with lysis buffer to 15 U/ml. At this point, three other cycles of sonication were performed to obtain an average DNA fragment size between 200 and 500 bp. The precipitation step was performed by incubating samples (250 μl each) 15 min on ice after adding 0.1 vol of 2.5 M KCl. Proteins were then precipitated by centrifugating for 3 min at 1200*g* and 4°C; then, pellets were washed five times with 0.25 M KCl and lastly resuspended in 500 μl of 1× TE buffer [10 mM tris-HCl (pH 8) and 1 mM EDTA). Resuspended samples, in parallel with 50 μl of not precipitated chromatin (INPUT), were digested by adding proteinase K (0.5 mg/ml) and incubating them for 3 hours at 56°C. DNA was subsequently purified with phenol/chloroform extraction, followed by ethanol precipitation and pellets resuspended in 40 μl of 1× TE buffer. Specific DNA fragments recovered in the K^+^/SDS precipitates were determined by real-time PCR.

### END-seq technique

END-seq procedure has been strictly performed as previously published and carefully detailed in (*71*). Spike-in pre–B cells were treated for 24 hours with imatinib (3 μM; Selleckchem, #S2475) and then for additional 24 hours with imatinib and doxycycline (3 μg/ml; Merck, #D9891), before detaching cells. HCT116 cells were treated or not with 10 μM CPT (Merck, #C9911) for 10 or 20 min and successively detached from cell plate by trypsin. A total of 8 × 10^6^ HCT116 cells and 6 × 10^5^ pre–B cells were washed with PBS and embedded in each agarose plug (Bio-Rad CHEF Mammalian Genomic DNA plug kit, #170-3591). All steps are performed in plugs to minimize externally produced DNA damage. Two to four plugs were produced for every cellular condition for each experiment. Plugs were allowed to solidify at 4°C and were then incubated with proteinase K solution (Puregene, QIAGEN, #158920) for 1 hour at 50°C and then O/N at 37°C. The next day, proteinase K was removed, and plugs were washed in buffer containing 10 mM tris (pH 8.0) and 50 mM EDTA (wash buffer) and then in a buffer containing 10 mM tris (pH 8.0) and 1 mM EDTA (TE buffer). Plugs were then treated with RNase A (Puregene, QIAGEN, #158924) for 1 hour at 37°C and washed again in wash buffer. Plugs are then stored at 4°C until the next day. Plugs are treated with exonuclease VII (NEB, #M0379L), exonuclease T (NEB, #M0265L), and Klenow fragment (NEB, #M0212L) with accurate washing between indicated steps. END-seq adapter 1 (IDT; see below for sequence) was then ligated using Quick ligase (NEB Quick Ligation Kit, #M2200L). After an O/N washing step, DNA was recovered from plug by agarose melting and β-agarase I digestion (NEB, #M0392L) and lastly dialized using dialysis membrane (0.1 μm; Millipore, #VCWP04700). After proteinase K treatment, DNA was sheared to a median size of 300 bp by sonication with Bioruptor Sonicator (Diagenode) and ethanol-precipitated. Biotinylated DNA fragments were purified using Dynabeads MyOne Streptavidin C1 (Thermo Fisher Scientific, #65002). Recovered fragments were then end-repaired using T4 DNA polymerase (NEB, #M0203L), DNA Pol I Large Klenow fragment (NEB, #M0210L), and T4 polynucleotide kinase (NEB, #M0201L). Last, fragments were A-tailed with Klenow fragment (NEB, # 2 (IDT; see Table 1) using the NEB Quick Ligation Kit (#M2200L). Libraries were prepared by first digesting the hairpins on both adapters with USER enzyme (NEB, #M5505L) and PCR-amplified for 16 cycles using TruSeq index adapters (IDT; see Table 1). After purification of PCR products by AMPure XP beads (Beckman Coulter, #A63880) and agarose gel extraction (QIAquick Gel Extraction Kit, QIAGEN, #28706), all libraries were quality-controlled and quantified using Bioanalyzer, Qubit, and qPCR. Sequencing was performed on the Illumina HiSeq X, 150-bp paired-end reads (external service from Biodiversa S.r.l., Italy).

**Table 1.** Index codes for each sample.

Experiment	Sample	Index code
Experiment 1	Untreated cells	D701 + D501
	CPT 10 min	D701 + D502
	CPT 20 min	D702 + D502
Experiment 2	Untreated cells	D702 + D501
	CPT 10 min	D703 + D501
	CPT 20 min	D703 + D502

Sequences of adapters and primers used in END-seq protocol (“*” denotes a phosphothiorate bond): END-seq adapter 1, phosphate GATCGGAAGAGCGTCGTGTAGGGAAAGAGTGUU biotin-dT U biotin-dT UUACAC TCTTTCCCTACACGACGCTCTTCCGATC*T; END-seq adapter 2, phosphate GATCGGAAGAGCACACGTCUUUUUUUUAGACGTGTGCTCTTCCGATC*T; D501 TruSeq barcoded primer p5 [index 2 (i5) adapters], AATGATACGGCGACCACCGAGATCTACACTATAGCCTACACTCTTTCCCTACACGACGCTCTTCCGATC*T; D502 TruSeq barcoded primer p5 [index 2 (i5) Adapters], AATGATACGGCGACCACCGAGATCTACACATAGAGGCACACTCTTTCCCTACACGACGCTCTTCCGATC*T; D701 TruSeq barcoded primer p7 [index 1 (i7) adapters], CAAGCAGAAGACGGCATACGAGATATTACTCGGTGACTGGAGTTCAGACGTGTGCTCTTCCGATC*T; D702 TruSeq barcoded primer p7 [index 1 (i7) adapters], CAAGCAGAAGACGGCATACGAGATTCCGGAGAGTGACTGGAGTTCAGACGTGTGCTCTTCCGATC*T; D703 TruSeq barcoded primer p7 [index 1 (i7) adapters], CAAGCAGAAGACGGCATACGAGATCGCTCATTGTGACTGGAGTTCAGACGTGTGCTCTTCCGATC*T.

### Comet assay

Neutral and alkaline comet assays have been performed with standard procedures as previously detailed in (*72*). HCT116 cells were detached from cell plate, and a cell suspension of 10^5^ cells/ml was treated with 10 μM CPT (Merck, #C9911) for indicated times. 10 μl of cell solution is then combined with 100 μl of molten CometAssay LMAgarose at 37°C (Bio-techne, #4250-050-02) and immediately pipetted in 30 μl of aliquots into 20-well comet slides (Bio-techne, #4252-02 K-01). Slides were incubated for 30 min at 4°C in the dark to allow agarose gelling. Cells were lysed submerging slides in lysis solution (Bio-techne, #4250-010-01) for 1 hour at 4°C in the dark. Then the procedure changes depending on the type of assay. For alkaline comet, after draining the lysis buffer, slides were immersed in alkaline unwinding solution (200 mM NaOH and 1 mM EDTA) for 1 hour at 4°C in the dark. Slides were placed in electrophoresis slide tray, and voltage has been applied for 30 min at 21 V in CometAssay Electrophoresis System II (Bio-techne, #4250-050-ES) at 4°C. After washing in water and 70% ethanol, samples were dried at 37°C for 15 min.

For neutral comet, lysis buffer was drained, and slides were immersed in neutral electrophoresis buffer [100 mM tris (pH 9) and 300 mM sodium acetate] for 30 min at RT; the electrophoresis was performed at 4°C for 45 min (21 V). Slides were immersed in DNA precipitation solution (1 M ammonium acetate in 95% ethanol solution) for 30 min at RT in 70% ethanol for additional 30 min and lastly dried at 37°C for 15 min. For both procedures, dried agarose circles were stained using SYBR Gold Nucleic Acid Gel Stain (Thermo Fisher Scientific, #S11494) for 30 min at RT and lastly rinsed in water. Slides were visualized by Nikon Eclipse 90i Microscope. Tail moment and DNA in tail quantification analysis were performed using ImageJ OpenComet software.

### Western blot

Cells, collected and resuspended in Laemmli buffer (4% SDS, 20% glycerol, 0.125 M tris, and 1× protease inhibitor), were sonicated for 20 min with Bioruptor Sonicator (Diagenode) and quantified by Lowry method. After incubation at 100°C for 10 min, samples were loaded in Bolt 4 to 12% bis-tris plus (Thermo Fisher Scientific, #NW04120BOX) and transferred onto nitrocellulose membrane (Thermo Fisher Scientific, #88018). Proteins transfer was confirmed by Ponceau staining (RNase H1 Western blot) or by No-Stain Protein Labeling Reagent (Invitrogen, #A44449) according to the manufacturer’s instructions (TFIIS western blots). Membrane was blocked with tris-buffered saline (20 mM tris and 150 mM NaCl)–0.1% Tween and 5% BSA for 1 hour at RT and incubated with the following antibodies O/N at 4°C: anti-DYKDDDK antibody (1:1000; Cell Signaling Technology, #2368), anti-Top1 (1:1000; Santa Cruz Biotechnology, catalog no. sc-5342), and anti-TFIIS (1:1000; Abcam, ab185947). After three washes in tris-buffered saline–0.1% Tween, membrane was incubated for 1 hour at RT with secondary antibodies: anti-rabbit (Abcam, #ab205718), anti-mouse (Invitrogen, #sc2005), and anti-goat (Invitrogen, #sc2922). Membrane was captured by using enhanced chemiluminescence (Thermo Fisher Scientific, #32132) at ChemiDoc imaging system (Bio-Rad). Immunoblot of BRCA2 was carried out as described in (*69*).

### Micronucleus detection

For micronucleus detection assay, HeLa, U2OS or U2OS-RH, HEK293m, and HCT116 cells were seeded at a density of 2 × 10^5^ cells per well in 35-mm dishes containing a 24-mm by 24-mm cover glass. After 24 hours, cells were treated with 10 μM and/or 25 μM drugs in fresh medium for 60 min. To distinguish cells in the S phase, cells were incubated with 10 μM EdU 30 min before, during the treatment, and 1 hour later for a total of 2.5 hours. To determine cell cycle subpopulations other than S phase, immediately after drug administration and EdU incubation, cells were washed with 1× PBS and incubated with 10 μM BrdU (Thermo Fisher Scientific, #B23151) solution for further 2.5 hours. Upon drug and EdU/BrdU administration, cells were leaved in drug-free medium for further 24 hours for micronucleus detection at the next mitosis (although CPT induces S phase blockage, 24 hours of recovery ensures that ~90% of cells have passed through the cell division phase). At this point, cells were fixed with formaldehyde 4% in 1× PBS for 15 min at room RT, washed twice with 3% BSA in 1× PBS (3% BSA/PBS), and permeabilized by 20 min incubation in 0.5% Triton X-100 in 1× PBS at RT. Cells were then washed once with 3% BSA/PBS, and EdU detection was performed by Click-iT EdU Assay (Thermo Fisher Scientific, #C10339). After 30 min of incubation at RT, the reaction cocktail was removed, and slides were washed with 1 ml of 3% BSA/PBS. Last, cells were incubated with a 4′,6-diamidino-2-phenylindole (DAPI) solution (3.3 ng/μl in water) for 30 min and then washed with water before being mounted with Mowiol upside down on microscope slides.

For BrdU staining, cells were denatured immediately before EdU staining. Denaturation protocol consists in 8 min of incubation with 4 N of HCl at 25°C, two washing steps (5 min each) with 1× PBS, 5 min of incubation in phosphate/citric acid (pH 7.4) (0.2 M Na_2_HPO_4_ and 0.1 M citric acid) and four more washing steps (two with 1× PBS and two with 3% BSA/PBS). In this case, after denaturation and EdU staining, cells were blocked with 3% BSA/PBS for 1 hour at RT and subsequently incubated O/N at 4°C with an anti-BrdU antibody (Thermo Fisher Scientific, #B35130) diluted 1:200 in blocking buffer plus 0.1% Triton X-100. Cells were then incubated with anti-mouse Alexa Fluor 488 secondary antibody (Thermo Fisher Scientific, #A11001) diluted 1:500 in 3% BSA/PBS for 1 hour at RT. After incubation, slides were washed with 3% BSA/PBS and incubated with a DAPI solution (3.3 ng/μl in water) for 30 min. A final washing step in water was performed before mounting slides with Mowiol upside down on microscope slides.

When micronucleus analysis was performed in cells overexpressing RNase H1, the overexpression of the protein was assessed by immunofluorescence. For HeLa cells transfected with pcDNA3–RNase H1 plasmid, cells were immunolabeled for hemagglutinin-tag before BrdU staining. Cells were incubated for 1 hour with an anti–hemagglutinin-tag antibody (Cell Signaling Technology, #3724) diluted 1:1000 in 3% BSA/PBS at RT. After washing in 3% BSA/PBS, cells were incubated for 1 hour with anti-rabbit Alexa Fluor 594 secondary antibody (Thermo Fisher Scientific, #A11037) diluted 1:1000 in 3% BSA/PBS. Cells were washed and then stained for BrdU detection.

For U2OS-RH cell line, CPT and EdU were administrated to cells previously induced with doxycycline for 48 hours. Fixation, permeabilization, and EdU detection were performed as described above. To detect RNase H1, a primary antibody against a FLAG-tag fused to the enzyme was used. Cells were incubated O/N at 4°C with anti-DYKDDDK antibody (Cell Signaling Technology, #2368) diluted 1:800 in blocking buffer. After washing with 1× PBS, cells were incubated with anti-rabbit Alexa Fluor 488 secondary antibody (Thermo Fisher Scientific, #A11008) in blocking buffer for 1 hour at RT. Next, DAPI staining and mounting were performed as described above.

For micronucleus detection in TFIISm-expressing HEK293 cell line, HEK293 cells were seeded at a density of 2 × 10^5^ cells per well in 35-mm dishes containing a 24-mm by 24-mm poly-l-lysine–coated cover glass. After treatment with doxycycline for 48 hours to induce TFIISm expression, 10 or 25 μM CPT and EdU/BrdU were administrated to cells, and immunofluorescence assay was performed as described above.

### Anaphase bridges and lagging chromosome detection

For the detection of anaphase bridges and lagging chromosomes, HeLa, U2OS, or U2OS-RH cells were seeded at a density of 1 × 10^6^ cells in T25 flasks. After 24 hours, cells were treated with 10 μM CPT in fresh medium for 60 min and labeled with EdU as previously described. After EdU administration, cells were incubated for 20 hours with nocodazole (50 ng/ml; Merck, #M1404). At the end, G_2_-M cells were collected by mitotic shake-off and released in fresh medium for further 50 min in 35-mm dishes containing a 24-mm by 24-mm poly-l-lysine–coated cover glass. At this point, anaphase cells were fixed for 15 min with PTEMF buffer [20 mM Pipes (pH 6.8), 1 mM MgCl_2_, 10 mM EGTA, 0.4% Triton X-100, and 8% paraformaldehyde) at RT, washed with 1× PBS, and then blocked with 3% BSA in 1× PBST (1× PBS and 0.5% Triton X-100) at 4°C O/N. Primary antibody incubation was performed O/N at 4°C using an anti-PICH (Millipore, #04-1540) or an anti-RPA70 antibody (Abcam, #ab79398) respectively diluted 1:100 and 1:750 in blocking buffer. Anti-mouse Alexa Fluor 488 secondary antibody (Thermo Fisher Scientific, #A11001) was used for secondary antibody incubation (1 hour) and diluted 1:1000 in blocking buffer. After primary or secondary antibodies incubation, slides were washed with 3% BSA/PBS. DAPI staining was then performed as previously described.

### γH2AX detection

For γH2AX detection, HeLa cells were seeded at a density of 2 × 10^5^ cells per well in 35-mm dishes containing a 24-mm by 24-mm cover glass. After 24 hours, cells were treated with 10 μM CPT in fresh medium for 60 min. Cells were fixed immediately after EdU (and BrdU, where indicated) administration. EdU staining protocol was performed as described above. Cells were then blocked for 30 min with 8% BSA/PBS at RT (gently rocking) and incubated for 2 hours at RT with anti-γH2AX antibody (Millipore, #05-636) diluted 1:1000 in 1% BSA/PBS. After primary antibody incubation, slides were washed with 1× PBS and incubated for 1 hour with anti-mouse Alexa Fluor 488 secondary antibody (Thermo Fisher Scientific, #A11001) diluted 1:1000 in the same buffer used for primary antibody. In case of EdU/BrdU dual labeling, Alexa Fluor 647 azide (Thermo Fisher Scientific, #A10277) was used instead of Alexa Fluor 594 azide included in the Click-iT EdU Assay kit. BrdU staining was performed after γH2AX staining by using only primary antibody conjugated with Alexa Fluor 488. DAPI staining was then performed as previously described.

### pATM and pATR detection

For S1981-phospjorylated ATM (pATM) and S428-phosphorylated ATR (pATR) detection, 2.5 × 10^5^ of TFIISm-expressing HEK293 cells were seeded in 35-mm dishes containing a 24-mm by 24-mm cover glass. After TFIISm induction, cells were treated with 10 μM CPT in fresh medium for 10 and 60 min. At this point, cells were fixed with 4% formaldehyde in 1× PBS for 15 min at RT, washed twice with 1× PBS, and permeabilized by a 20-min incubation in 0.5% Triton X-100 in 1× PBS at RT. Cells were then blocked for 1 hour with 1% BSA/PBS and 5% BSA/PBS, respectively, for pATM and pATR staining at RT (gently rocking). After permeabilization, cells were incubated for 2 hours at RT with anti-pATM (S1981) (Santa Cruz Biotechnology, sc-47739) or anti-pATR (S428) (Cell Signaling Technology, #2853) diluted 1:250 and 1:100, respectively, in blocking buffer. Slides were then washed with 0.1% PBST (0.1% Tween 20 in 1× PBS) and incubated for 1 hour with anti-mouse Alexa Fluor 488 secondary antibody (Thermo Fisher Scientific, #A11001) or anti-rabbit Alexa Fluor 594 secondary antibody (Thermo Fisher Scientific, #A11037) diluted 1:1000 in the same buffer used for primary antibody. After three washes with 0.1% PBST, DAPI staining was performed as previously described. Cells were observed with Nikon A1R Confocal Microscope (Eclipse Ti2), and pATM/pATR mean fluorescence was quantified using ImageJ software.

### Image analysis and representation

Fluorescent images, if not differently specified, were acquired by using fluorescence microscope Eclipse 90i (Nikon) and then analyzed with the ImageJ software. Cells were classified in S phase or non–S phase according to their positivity to EdU staining. In case of EdU/BrdU dual labeling, cells were divided into three main classes: G_1_ or late G_1_ phase when only BrdU was incorporated; late G_1_ or S phase as cells entering S phase incorporated both thymidine analogs; and late S or early G_2_ phase when only EdU was incorporated. Cells that were not replicating during either pulse were conegative for both EdU and BrdU staining.

To perform micronucleus or bridge analysis, micronuclei and bridges were counted for each captured image. As already done for cells, micronuclei and bridges were also classified in EdU^+^/EdU^−^ and BrdU^+^/BrdU^−^ according to their positivity to EdU and BrdU staining. Micronucleus/bridge data were reported as number of micronuclei/bridges per 100 cells for each cell class and normalized or not with respect to the untreated control. For γH2AX, pATM, and pATR signal, fluorescence mean value was measured for each cell and background-subtracted. In addition, γH2AX signal increase was evaluated considering the different classes of cells previously described. Data were plotted using GraphPad Prism version 9. Statistical analysis was carried out using GraphPad Prism 9.

### Proximity ligation assay

PLA was carried out by the Duolink In Situ PLA Kit (Merck, #DUO92101-1KT) according to datasheet instructions. Briefly, 2 × 10^5^ of HCT116 cells were seeded in 35-mm dishes containing an 18-mm by 18-mm glass slide. After 24 hours, cells were treated with 10 μM CPT in fresh medium for 5 min and fixed with 4% formaldehyde in 1× PBS for 15 min at RT. After washing with 1× PBS, cells were permeabilized by incubating them with 0.1% Triton X-100 in 1× PBS three times for 5 min at RT (gently rocking). Anti-Top1cc (Merck, #MABE1084) and anti–tag-V5 (Abcam, #ab15828) antibodies were diluted 1:2000 and 1:3000, respectively, and incubation was performed O/N at 4°C. DAPI staining was performed as described in the “Micronucleus detection” section. For PCNA and RNAPII PLA assay, 2 × 10^5^ of TFIISm-expressing HEK293 cells were seeded in 35-mm dishes containing an 18-mm by 18-mm poly-l-lysine–coated glass slide. After TFIISm induction, cells were treated with 10 μM CPT in fresh medium for 10 and 60 min. For transcription or replication inhibition, cells were treated with 1 μM triptolide, cordycepin (12.5 μg/ml), or 1 μM aphidicolin for 2 hours prior the 10-min CPT treatment. Fixation and permeabilization were performed as described above. Anti-PCNA (PC11; sc-53407) and anti-RNAPII (H-224; sc-9001) antibodies were diluted 1:100, and incubation was performed O/N at 4°C. All the incubations were done by putting each slide upside down on 40 μl of each solution previously placed on a parafilm layer. Cells were observed with Nikon A1R Confocal Microscope (Eclipse Ti2), and the number of PLA foci was quantified using ImageJ software (analysis protocol available at https://microscopy.duke.edu/guides/count-nuclear-foci-ImageJ).

### Bioinformatic analysis

#### DRIP-seq analysis

DRIP-seq libraries were quality checked using Fastqc, and reads were trimmed using Cutadapt (*73*). Reads were aligned on both human genome (hg19) and spike-in yeast genome (sacCer3) using Burrow-Wheeler Aligner (*74*). Alignment sorting, filtering for ENCODE blacklist (*75*) region, and duplicated reads removal were performed using SAMtools (*76*) and Picard (table S1). Peak calling and genomic signal computation for each library were performed using macs2 using paired IP-Input libraries as -t and -c, respectively (*77*). Only peaks that were present in both replicates for at least one biological condition were considered for further analysis. Read counts computation for each peak was performed using bedtools (*78*) *multicov* command. Reads counts were normalized on the base of library dimension and percentage of spike-in reads in each library.

Differential analysis was performed with limma R (version 2.30.0) library (*79*). First, we performed a comparison between DRIP-seq libraries of immunoprecipitated R loop– and RNase H1–treated libraries to select only true-positive R loop regions. Then, we performed differential analysis between each treatment condition and control. DRIP-seq signal tracks for each condition were created using deepTools *bamCoverage* function, using the normalization scaling factor computed using both library dimension and spike-in fraction for each library. DRIP-seq peak annotation was performed using DROPA (*80*) with standard settings. Lamin-associated domain coordinates were downloaded from UCSC table browser. R loop peak distance from lamina-associated domains was performed using bedtools *closest* command.

Genomic mapping for Top1-seq, Top1 ChIP-seq, RNAPII ChIP-seq, GRO-seq, and SNS-seq were downloaded as processed and normalized (as in figure legends) bigwig and Wig files. Top1cc levels (Top1-seq), Top1 ChIP-seq, and RNAPII ChIP-seq Wig files were converted to bigWig files using *rtracklayer* R library (*81*). To calculate read counts of Top1cc over R loop peaks, Top1-seq libraries were mapped to hg19 human genome using bowtie2 aligner, and read counts computation was performed using bedtools *multicov* command. Plots of DRIP-seq, Top1-seq, Top1 ChIP-seq, RNAPII ChIP-seq, GRO-seq, and SNS-seq levels over genomic features were performed using deepTools (*82*) *computeMatrix* and *plotHeatmap* functions. TAD boundaries and replication IZs were converted from hg38 to hg19 human genome version using liftOver UCSC tool. Enrichment analysis of R loop regions over TAD boundaries, replication IZs, and END-seq peaks was performed using the command bedtools *shuffle* and *intersect* commands (100 times), and then we calculated mean log_2_(fold change) and −log_10_(*P* values) of observed/expected values for each and R loop class, using a binomial test. Barplots of enrichment were made in R (version 4.1.3) using “ggplot2” (version 3.3.6).

#### END-seq data analysis

Adapter sequences of paired end reads in fastq format were trimmed, using Trim Galore (version 0.6.5), and then reads were aligned to hg19 reference human genome and to mm10 reference mouse genome, using Bowtie2 with default settings (version 2.5.0). Only properly paired reads with mapping quality (MAPQ) ≥ 30 were kept, including duplicated reads, using SAMtools (version 1.16.1) (*83*) (table S1). Prior peak calling–aligned paired-end reads in BAM format were splitted according to first-in pair orientation to discriminate reads aligned on the left side (F2R1, reverse-minus strand) and on the right side (F1R2, forward-plus strand) respect with the DSB. Peak calling was performed using MACS2 (version 2.2.7.1) (*77*) and using control samples as “input.” Peak calling was performed separately for each replicate and according to first-in-pair reads orientation, using the parameters --nomodel --nolambda -q 0.05 --keep-dup all -f BAMPE as previously described in (*84*).

After peak calling, to specifically identify forward and reverse peaks resembling the whole END-seq signal, we searched for the closest forward peaks respect with the reverse ones, using bedtools *closest* (version 2.30.0) (*78*) and discarded pair of peaks resulting in negative values of distance or positive values of distance above or equal to 150 bp. Then, we retrieved start coordinates of reverse peaks and end coordinates of the closest forward peaks to obtain whole END-seq peaks for each replicate.

To normalize target genome reads on spike-in genome reads covering “on-target” locus (TCRβ), as indicated in (*48*), we firstly created a universe of peaks that were present in both replicates for each condition, excluding ones overlapping hg19-blacklisted regions (*85*) and obtained reads covering peaks (RiP) using bedtools *multicov* (version 2.30.0) (*78*). On-target spike-in RiP was divided by quality-filtered reads (RQC) and then by the minimum resulting factor within each replicate. The resulting spike-in scaling factors were then multiplied by a scaling factor performed on library size to lastly normalize target genome reads.

Differential expression analysis for each condition (treated versus control) was performed using R/Bioconductor package “limma” (version 3.50.3) (*79*) with default settings, and peaks with log_2_(fold change) of >1 or log_2_(fold change) of <−1 and *P* <0.05 were considered as differentially expressed. Volcano plot of differentially expressed peaks was made with “EnhancedVolcano” R/Bioconductor package (version 1.12.0). Then, as we were interested in regions significantly enriched in treated over control samples, the following steps only regard positively enriched END-seq peaks. First, END-seq peaks that resulted differentially expressed in both 10′ CPT and 20′ CPT treatment samples were included in the “persistent” class (*n* = 3498) to further distinguish peaks that were exclusively enriched at 10′ CPT (“transient,” *n* = 763) from ones enriched at 20′ CPT (“late,” *n* = 948). Second, within each class, overlapping peaks were merged to obtain clusters of DSB. Successively, we also considered as persistent all those transient and late clusters that were overlapping each other (*n* = 273) to further refine kinetic classes of DSB clusters.

BigWig files of END-seq signal scores for control and treated conditions were obtained from BAM files using *bamCoverage* command from deepTools (version 3.5.1) (*82*) normalizing by the previously calculated scaling factors, and then mean signal of replicates for each condition was obtained using bigwigCompare (version 3.5.1) (*82*). BigWig files of plus and minus strand signal were obtained from aligned paired-end reads, using samtools view (version 1.16.1) (*83*), command and -f option to include right and left alignments matching SAM flags for forward or reverse reads according to first-in pair orientation, and then using samtools merge (version 1.16.1) (*83*), the outputs were merged to obtain a single BAM file for plus and minus strand alignments. Metaplots of END-seq signal scores per genomic regions were performed using computeMatrix and plotHeatmap tools from deepTools (version 3.5.1) (*82*). Bigwigs of histone markers ChIP-seq and DNase-seq in HCT116 cells for the hg19 assembly were obtained from ENCODE website as processed and normalized tracks. Proportion and enrichment of END-seq peaks over gene features plots were performed using DROPA tool (version 1.0.0) (*80*) in GRCh37 Ensembl annotation. Randomization and over R loop regions of END-seq peaks were performed using the command bedtools *shuffle* (version 2.30.0) (*78*) (100 times), and then we calculated log_2_(fold change) and −log_10_(*P* values) of observed/expected values for each END-seq and R loop class, using a binomial test. Barplots of enrichment were made in R (version 4.1.3) (www.R-project.org) using ggplot2 (version 3.3.6).

To identify seDSBs, we analyzed separately peaks according to first-in pair orientation. First, we selected peaks present in both replicates within each condition using bedtools *intersect* and merged them using bedtools *merge*. Then, we identified forward and reverse peaks distant more than 150 bp from one another to retrieve “solitary” peaks ascribable to seDSBs. Reads covering peaks were calculated for both universes of peaks and then normalized, as previously described, distinguishing for forward and reverse reads for both spike-in and library scaling factors. Then, to remove potential outliers, only counts within the first and the third quartile were retained for the differential expression analysis. Differential expression analysis was performed as previously described, and persistent seDSB was defined as enriched peaks under both condition of treatment versus control condition. Visualization of genomic distribution of DRIP-seq, END-seq, and ChIP-seq data was performed using Integrative Genomics Viewer software (*86*).

### Statistical analysis

Statistical analyses were performed in Prism v9 (GraphPad Software) using tests shown in each figure legends. All the used analysis are hereafter reported: one-tailed ratio paired *t* test, multiple unpaired *t* test, one-tailed paired *t* test, two-tailed Mann-Whitney test, the nonparametric Mann-Whitney rank sum test, and the parametric paired *t* test: *P < 0.05; **P < 0.01; ***P < 0.001; ****P < 0.0001.

*Note added in proof*: After the manuscript was accepted for publication, the authors alerted the Editorial Office to an additional paper related to their data discussion:

K. B. Ynag, A. Rasouly, V. Epshtein, C. Martinez, T. Nguyen, I. Shamovsky, E. Nudler, Persistence of backtracking by human RNA polymerase II. *Mol Cell.* **84**, 897–909 (2024).

## Supplementary Materials

### This PDF file includes:

Figs. S1 to S9 Legend for table S1 Table S2 References
