# Supplementary material for: Human DNA topoisomerase I poisoning causes R loop–mediated genome instability attenuated by transcription factor IIS
Source: Sci Adv. 2024 May 24;10(21):eadm8196. doi: 10.1126/sciadv.adm8196 (PMC11122683; doi:10.1126/sciadv.adm8196)
Supplement: Supplementary file 1 — Figs. S1 to S9 Legend for table S1 Table S2 References [file sciadv.adm8196_sm.pdf]

Science Advances
AAAS

Supplementary Materials for

Human DNA topoisomerase I poisoning causes R loop–mediated genome instability attenuated by transcription factor IIS

Renée C. Duardo *et al.*

Corresponding author: Giovanni Capranico, giovanni.capranico@unibo.it

Sci. Adv. **10**, eadm8196 (2024)
DOI: 10.1126/sciadv.adm8196

The PDF file includes:

Figs. S1 to S9
Legend for table S1
Table S2
References

Other Supplementary Material for this manuscript includes the following:

Table S1

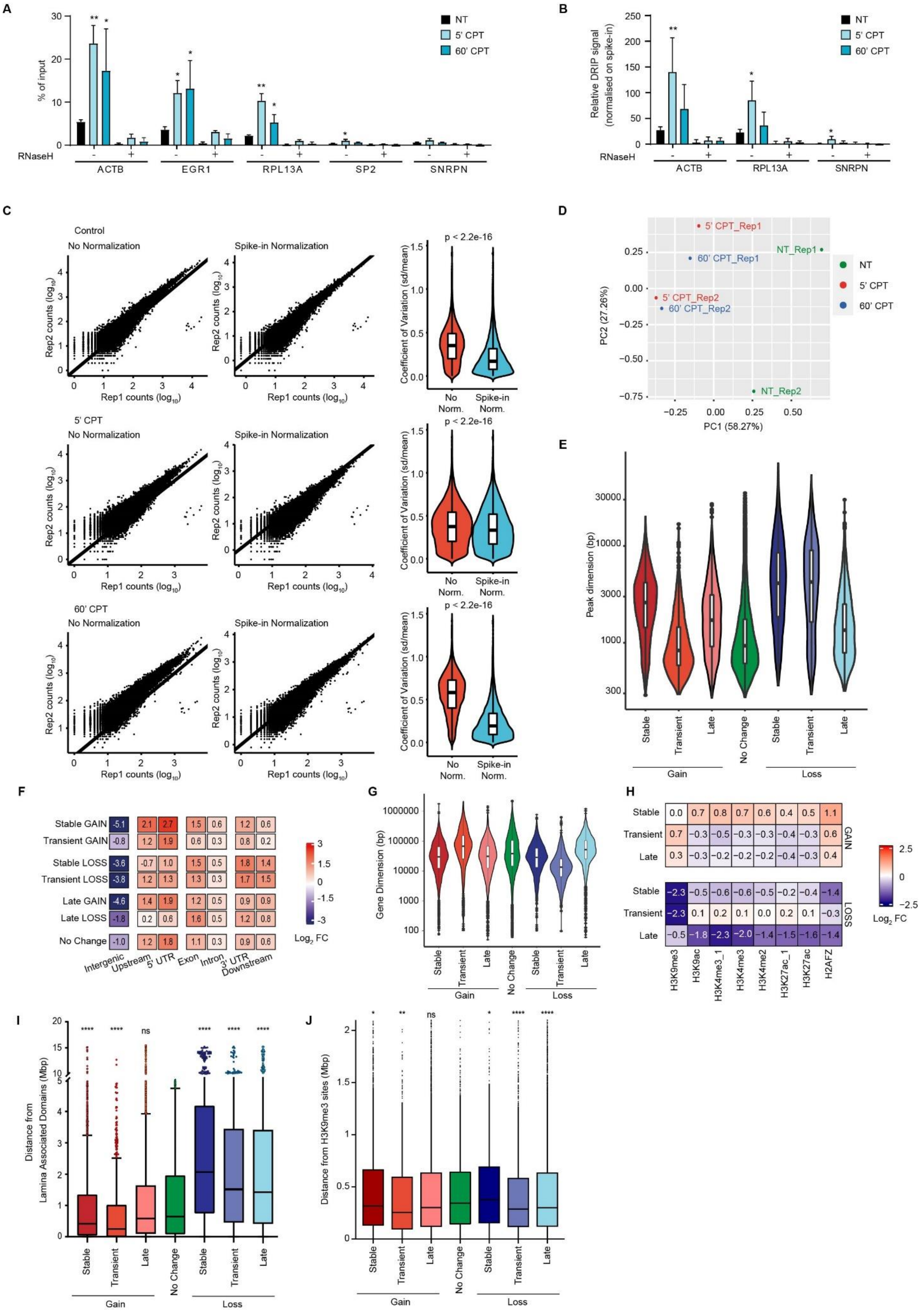

Figure S1. R-loop detection by DRIP, and genomic analyses of Top1cc-altered peaks. A. DRIP-qPCR assay after treating HeLa cells with 10 μM camptothecin for indicated times. During DRIP procedure, genomic DNAs were treated or not with RNaseH1 before immunoprecipitation with S9.6 Ab to demonstrate specific recovery of DNA-RNA hybrids. Each bar represents mean values ± SEM and statistical tests were performed with one-tailed ratio paired *t* test. Asterisks show p-values as follows: ∗, <0.05; ∗∗, <0.01; ∗∗∗, <0.001; and ∗∗∗∗, <0.0001. At least three biological replicates are reported. **B.** Same as in (A) but in HCT116 cell line. Values are normalized on *Saccharomyces cerevisiae spike-in* (PDC1 locus). **C.** Left, scatterplots of read counts reporting the correlation between the two biological replicates (Rep1, *x*-axis and Rep2, *y*-axis) of untreated human HCT116 cancer cells (NT), HCT116 cells treated for 5 minutes with 10 μM camptothecin (5' CPT) and HCT116 cells treated for 60 minutes with 10 μM camptothecin (60' CPT) with and without *spike-in* normalization as indicated. Read counts are $\log_{10}$ scaled. Right: violin plots showing the distribution of the coefficients of variation (*y*-axis) for NT, 5' CPT and 60' CPT DRIP-Seq counts with and without *spike-in* normalization (*x*-axis). Color code as in legend. Wilcoxon test p-values are reported. **D.** Principal Component Analysis (PCA) of normalized NT, 5' CPT and 60' CPT DRIP-Seq counts for the two biological replicates, Rep1 and Rep2. Color code as in legend. **E.** Violin plots showing distribution of DRIP peak dimension (*y*-axis) for each DRIP peak category (*x*-axis). **F.** Heatmap of DRIP peak enrichment (log2 ratio between observed peaks and expected by chance peaks) for each DRIP peak category (rows) in different genomic regions (columns). Numbers in each cell indicate fold-change values. **G.** Violin plots showing distribution of DRIP peak-annotated gene dimension (*y*-axis) for each DRIP peak category (*x*-axis). **H.** Heatmaps of enrichment of different histone marks at the observed DRIP peak categories (HCT116 cells, data from ENCODE database. Color legend represents the $\log_2$ fold-change over the expected values after DRIP peak randomization in all R-loop-associated regions. Numbers in each cell indicate fold-change values. **I.** Boxplots showing distributions of DRIP peak distance from LADs (Lamina Associated Domains) (*y*-axis) for each DRIP peak category (*x*-axis). Asterisks show p-values of Mann-Whitney test against "No Change" distribution as follows: ∗, <0.05; ∗∗, <0.01; ∗∗∗, <0.001; and ∗∗∗∗, <0.0001. **J.** Boxplots showing distributions of DRIP peak distance from H3K9me3 sites (*y*-axis, HCT116 cells, data from ENCODE database for each DRIP peak category (*x*-axis). Asterisks show p-values of Mann-Whitney test against "No Change" distribution as follows: ∗, <0.05; ∗∗, <0.01; ∗∗∗, <0.001; and ∗∗∗∗, <0.0001.

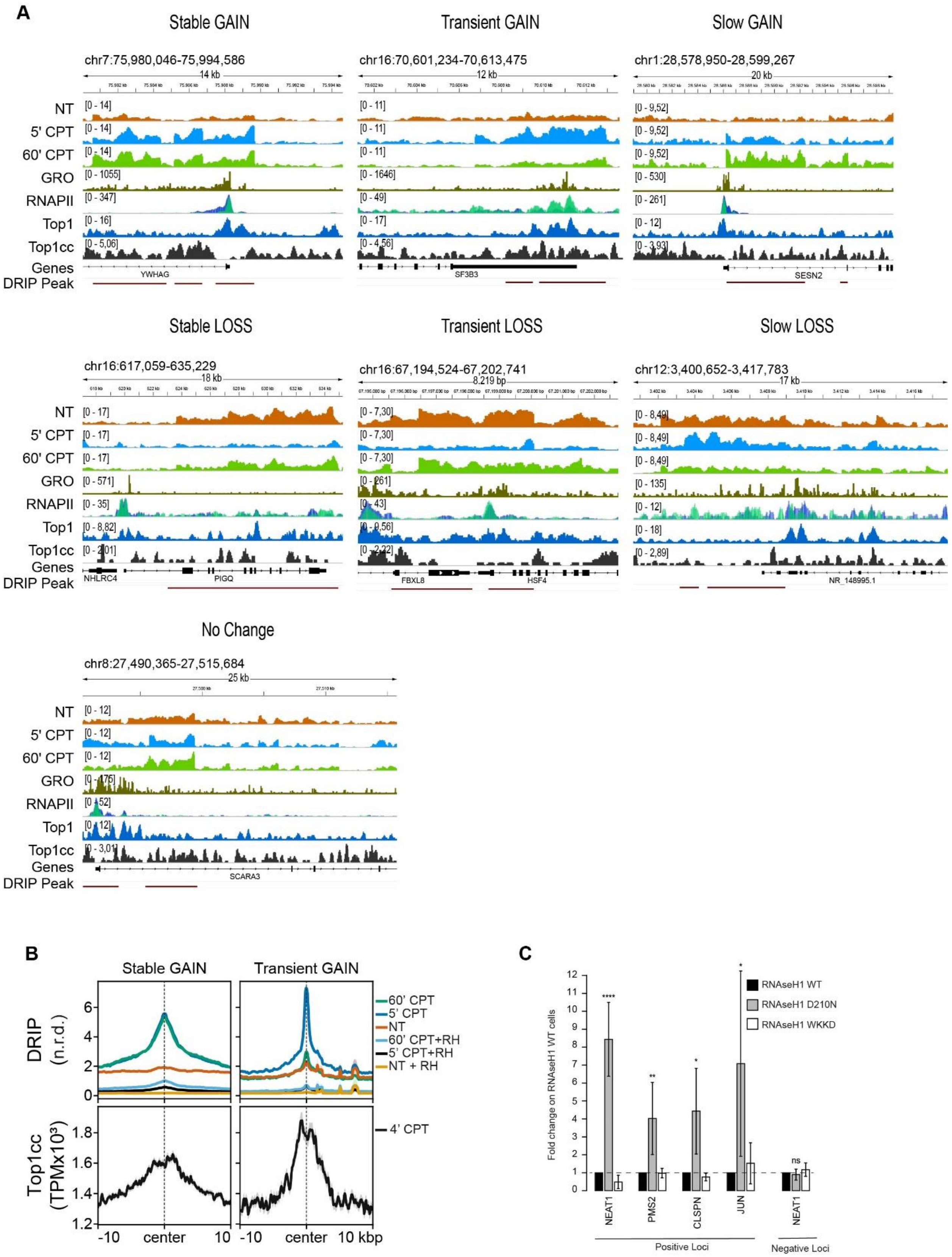

Figure S2. Representative genomic loci of hybrid categories. A. Integrative Genomics Viewer (IGV) screenshots of DRIP-seq (NT, 5' CPT and 10' CPT), GRO-seq, RNAPII ChIP-seq

(combined tracks for NT in green and for 10' CPT in blue), Top1 ChIP-seq and Top1cc-seq levels at representative genomic loci for each R-loop kinetic category. as reported in Figure 1D. DRIP peak row indicate the peak region. **B.** Metaplots of DRIP-seq and Top1cc-seq normalize levels at Stable and Transient R-loop GAIN regions of genes. "Center" represent the R-loop peak center, in a window of -/+ 10 kbp. Line colors as in legend. **C.** Hybrid detection with the R-ChIP method at four R-loop positive loci and a negative on. Bars represent at least 3 biological replicates of untreated cells and are normalized on HeLa cells stably overexpressing WT RNaseH1 protein (black bar). As expected, data show a specific R-loop recovery only in cells overexpressing D210N mutant RNaseH1. Color code as in the legend.

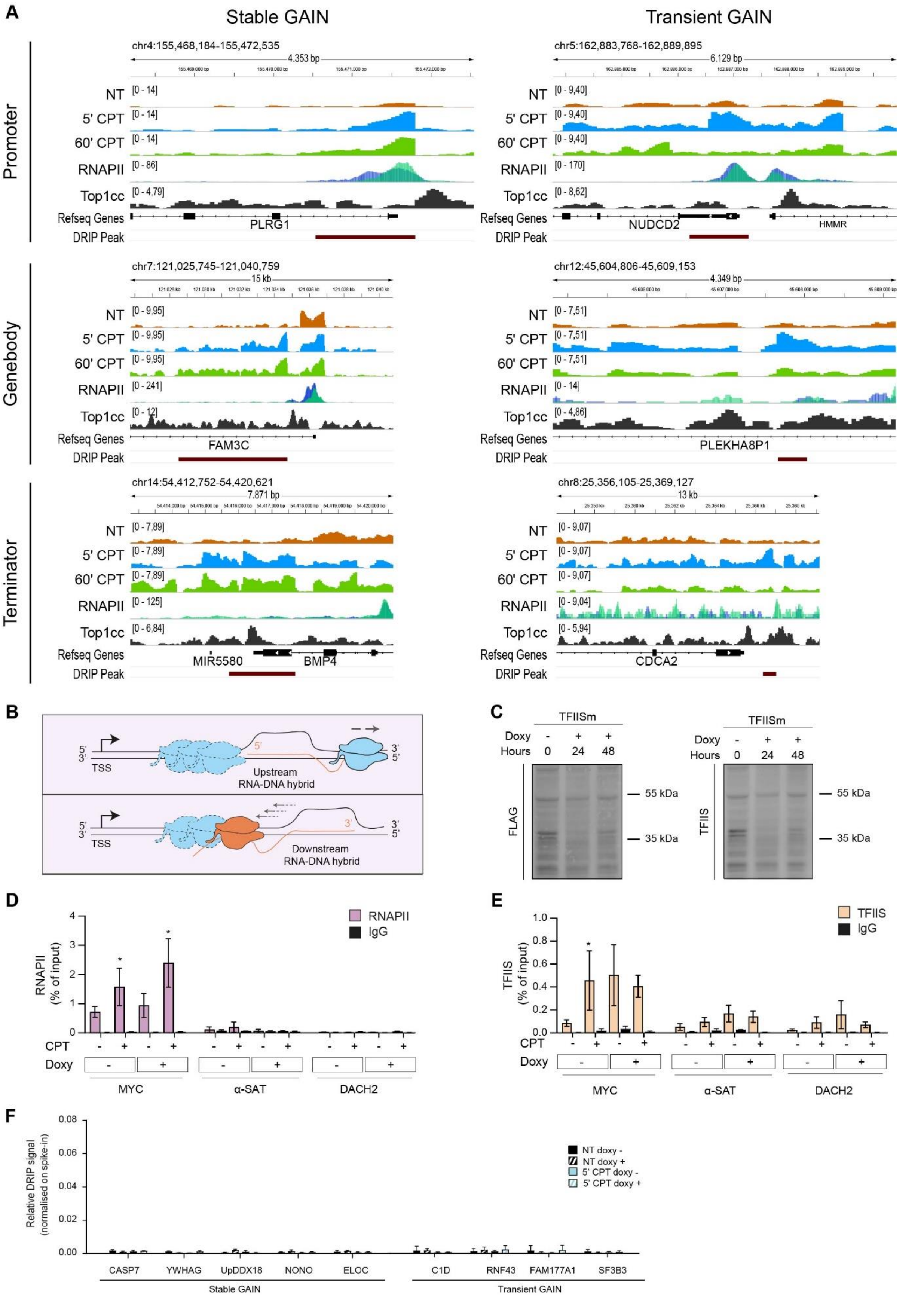
A
Stable GAIN
Transient GAIN
Promoter
Genebody
Terminator
chr4:155,468,184-155,472,535
4.353 bp
chr5:162,883,768-162,889,895
6.129 bp
chr7:121,025,745-121,040,759
15 kb
chr12:45,604,806-45,609,153
4.349 bp
chr14:54,412,752-54,420,621
7.871 bp
chr8:25,356,105-25,369,127
13 kb
NT
5' CPT
60' CPT
RNAPII
Top1cc
Refseq Genes
DRIP Peak
[0 - 14]
[0 - 86]
[0 - 4,79]
[0 - 9,40]
[0 - 170]
[0 - 8,62]
[0 - 9,95]
[0 - 241]
[0 - 12]
[0 - 7,51]
[0 - 14]
[0 - 4,86]
[0 - 7,89]
[0 - 125]
[0 - 6,84]
[0 - 9,07]
[0 - 9,04]
[0 - 5,94]
PLRG1
NUDCD2
HMMR
FAM3C
PLEKHA8P1
MIR5580
BMP4
CDCA2
B
5'
3'
TSS
Upstream
RNA-DNA hybrid
Downstream
RNA-DNA hybrid
C
TFIISm
Doxy
Hours
- + +
0 24 48
FLAG
TFIIS
55 kDa
35 kDa
D
RNAPII
IgG
RNAPII
(% of input)
CPT
Doxy
MYC
α-SAT
DACH2
E
TFIIS
IgG
TFIIS
(% of input)
F
Relative DRIP signal
(normalised on spike-in)
NT doxy -
NT doxy +
5' CPT doxy -
5' CPT doxy +
CASP7
YWHAG
UpDDX18
NONO
ELOC
C1D
RNF43
FAM177A1
SF3B3
Stable GAIN
Transient GAIN

Figure S3. RNAPII accumulation induced by short exposures of HEK293 cells to camptothecin.

A. Integrative Genomics Viewer (IGV) screenshots of DRIP-seq (NT, 5' CPT and 10' CPT), RNAPII ChIP-seq (combined tracks for NT in green and for 10'CPT in blue), and Top1cc-seq levels at representative genomic loci for stable and transient R-loop GAIN divided in promoter-associated (up to -10 kbp from promoter and 5'UTR), genebody-associated (located in exon and intron) and terminator associated (located in 3'UTR and Downstream 10 kbp) regions of genes, as reported in Figure 3A. DRIP peak row indicate the peak region. **B.** Possible mechanism of RNAPII accumulation and hybrid formation caused by Top1cc. **C.** Total protein staining as loading control for TFIISm expression induced by doxycycline (+). **D-E.** Bar plot of RNAPII and TFIIS levels at transcribed (MYC) or not transcribed (α-sat and DACH2) loci determined by ChIP (antibodies sc-47701 and ab185947, respectively). HEK293 cells were treated with CPT for 10 minutes. TFIISm was induced for 48 hours with doxycycline. DNA enrichment over input was quantified by real-time PCR. Data are from at least three biological replicates. Statistical analysis was performed using multiple unpaired t-test. Asterisks show p-values as follows: ∗, <0.05; ∗∗, <0.01; ∗∗∗, <0.001; and ∗∗∗∗, <0.0001. **F.** R-loop levels determined by DRIP-qPCR at indicated loci in not induced and induced TFIISm HEK293 cells treated with RNAseH1. DRIP enrichment over input was normalized on pFC53 plasmid *spike-in* (RF amplicon). At least three biological replicates are reported. Each bar represents mean values ± SEM and statistical tests were performed with one-tailed ratio paired *t* test. Asterisks show p-values as follows: ∗, <0.05; ∗∗, <0.01; ∗∗∗, <0.001; and ∗∗∗∗, <0.0001. At least three biological replicates are reported.

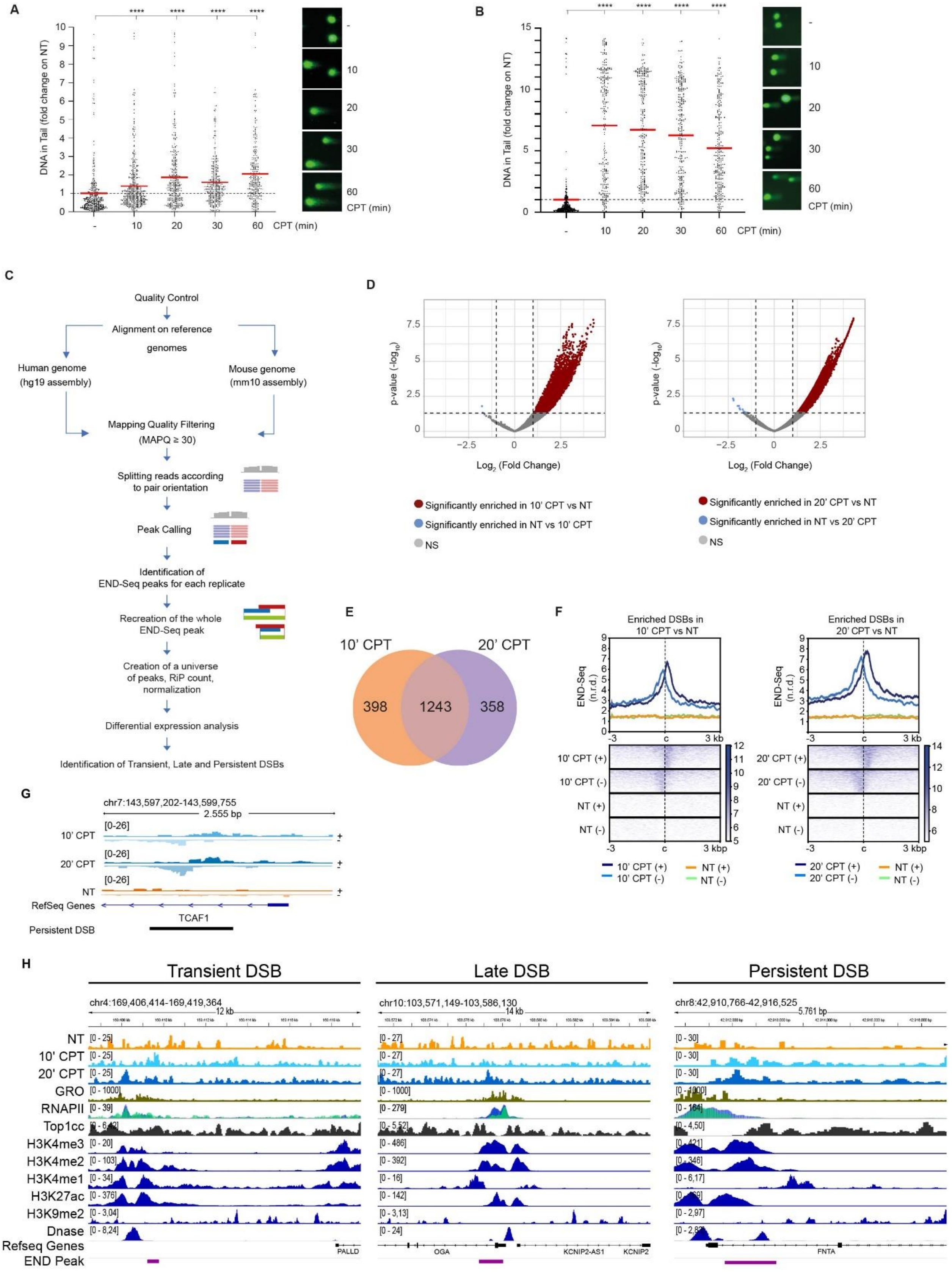
A
B
C
D
E
F
G
H
DNA in Tail (fold change on NT)
CPT (min)
Quality Control
Alignment on reference genomes
Human genome (hg19 assembly)
Mouse genome (mm10 assembly)
Mapping Quality Filtering (MAPQ ≥ 30)
Splitting reads according to pair orientation
Peak Calling
Identification of END-Seq peaks for each replicate
Recreation of the whole END-Seq peak
Creation of a universe of peaks, RiP count, normalization
Differential expression analysis
Identification of Transient, Late and Persistent DSBs
p-value (-log10)
Log2 (Fold Change)
Significantly enriched in 10' CPT vs NT
Significantly enriched in NT vs 10' CPT
NS
Significantly enriched in 20' CPT vs NT
Significantly enriched in NT vs 20' CPT
NS
10' CPT
20' CPT
398
1243
358
Enriched DSBs in 10' CPT vs NT
Enriched DSBs in 20' CPT vs NT
END-Seq (n.r.d.)
10' CPT (+)
10' CPT (-)
NT (+)
NT (-)
20' CPT (+)
20' CPT (-)
chr7:143,597,202-143,599,755
2.555 bp
RefSeq Genes
TCAF1
Persistent DSB
Transient DSB
Late DSB
Persistent DSB
chr4:169,406,414-169,419,364
chr10:103,571,149-103,586,130
chr8:42,910,766-42,916,525
NT
10' CPT
20' CPT
GRO
RNAPII
Top1cc
H3K4me3
H3K4me2
H3K4me1
H3K27ac
H3K9me2
Dnase
Refseq Genes
END Peak
PALLD
OGA
KCNIP2-AS1
KCNIP2
FNTA

Figure S4. DNA cleavage sites induced by short exposures of HCT116 cells to camptothecin. **A.** DSBs are detected in HCT116 cells after 10 minutes of 10 μM CPT and increase at 20 minutes by neutral Comet assay. At least 230 cells have been observed. Three biological replicates are reported. Statistical significance was calculated considering the distributions of treated samples *vs* untreated samples with the two-tailed Mann-Whitney test. *p* values are: ∗, <0.05; ∗∗, <0.01; ∗∗∗, <0.001; and ∗∗∗∗, <0.0001. Representative cell images are shown on the right. **B**. Alkaline Comet assay performed in HCT116 cells as in (A). At least 250 cells have been observed. Single-strand breaks are already at maximum levels after 10 minutes of treatment and can derive from Top1ccs. Three biological replicates are reported. Statistical significance as in (A). **C**. Workflow of analyses of END-Seq read sequences. After quality control, reads were aligned on human and mouse reference genomes and filtered for MAPQ. Aligned reads were then split for the first-in pair orientation (forward reads, mapping on the + strand, are reported in pink, while in violet are reported reverse reads, mapping on - strand). Peak calling has been performed separately for forward and reverse reads. Red and blue rectangles represent peaks for forward and reverse reads. After peak calling, forward and reverse peaks resembling the whole END-Seq signal were identified and then, start coordinates of reverse peaks (in blue) and end coordinates of the closest forward peaks (in red) were used to recreate the whole END-Seq peaks for each replicate (in green). After the creation of the universe of peaks, reads covering peaks were calculated and normalized. Then, a differential expression analysis was performed between treated and control conditions. **D.** Volcano plots of END-Seq peaks in 10' CPT *vs* NT (left) and 20' CPT *vs* NT (right) increased (red) or decreased (blue) in CPT-treated cells. Each dot represents an END-Seq peak. Colors as in legend. Vertical dotted lines indicate thresholds of $\log_2$(fold-change) > 1 and $\log_2$(fold-change) < -1 (*x*-axis). The horizontal dotted line indicates a threshold of p-value < 0.05, indicated as $-\log_{10}$(p-value) (*y*-axis). **E**. Venn diagram reporting numbers of DSBs after 10' CPT or 20' CPT treatment. Persistent DSB clusters are those in common between the two time points. **F.** Metaplots and heatmaps of END-Seq normalized signal of pair-oriented reads (+ strand, blue and green; - strand, light-blue and orange) at significantly higher DSB clusters after the two studied times: 10' CPT *vs* NT (left); 20' CPT *vs* NT (right). "c" indicates the center of DSB clusters. **G.** The genomic locus of a representative persistent DSB cluster reporting normalized read signals for 10' CPT (light blue), 20' CPT (blue) and NT (orange) samples on both plus (+) and minus (-) strands. **H.** Integrative Genomics Viewer (IGV) screenshots of END-seq (NT, 10' CPT and 20' CPT), GRO-seq, RNAPII ChIP-seq (combined tracks for NT in green and for 10'CPT in blue), Top1cc-seq, different histone marks ChIP-seq and DNase-seq levels at representative genomic loci for each DSB cluster kinetic category. as reported in Figure 4A,D and E. END peak row indicate the DSB cluster region.

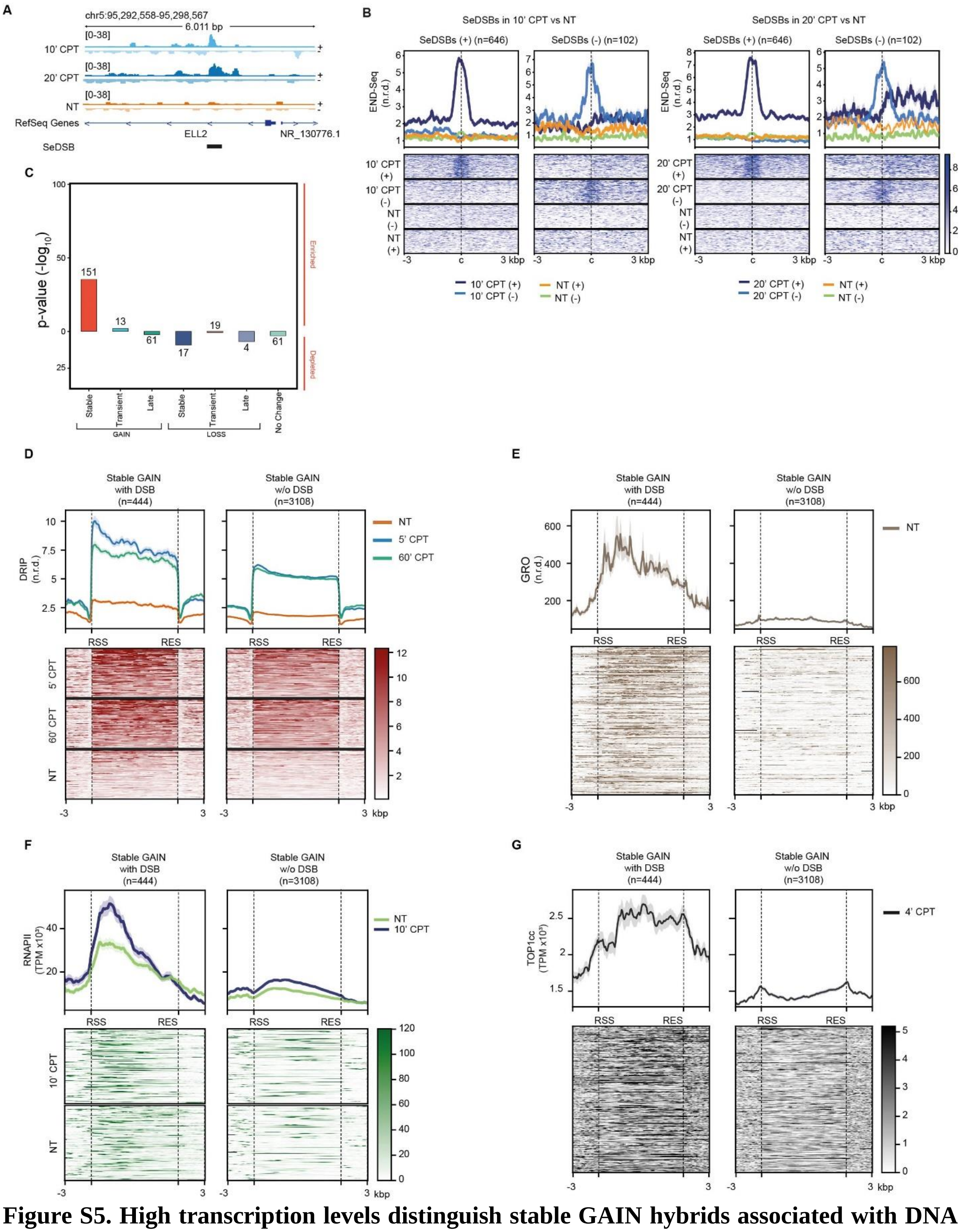

Figure S5. High transcription levels distinguish stable GAIN hybrids associated with DNA cleavage from those without DNA cleavage. A. The genomic locus of a representative persistent single-ended DSB reporting normalized read signals for 10' CPT (light blue), 20' CPT (blue) and NT (orange) samples on both plus (+) and minus (-) strands. **B.** Metaplots and heatmaps of END-

Seq normalized levels of pair-oriented reads (+ strands, blue and orange; - strand, light-blue and green) at significantly higher single-ended DSBs on the plus strand (+) and minus strand (-) at both 10' and 20' CPT treatments *vs* NT. "c" indicates the center of single-ended DSB regions. **C.** Enrichment analysis of observed seDSBs at DRIP categories vs. expected by genome randomization (n=100). Barplots report binomial test p-values (*y*-axis) of enrichment. $-\log_{10}$(p-value) are referred as "Enriched" for a positive overlap enrichment over expected, or "Depleted" for a depletion over expected. Numbers of observed overlaps at each category are reported. **D-G.** Metaplots (top) and heatmaps (bottom) of levels of DRIP-seq (D), GRO-seq (E), RNAPII ChIP-seq (F) and Top1cc-seq (G), at stable GAIN peaks with or without DSBs. RSS and RES represent the R-loop peak start site and the R-loop peak end site, respectively, in a window of -3/+3 kbp. Line colors as in legend. All signals are reported as normalized read-depth levels.

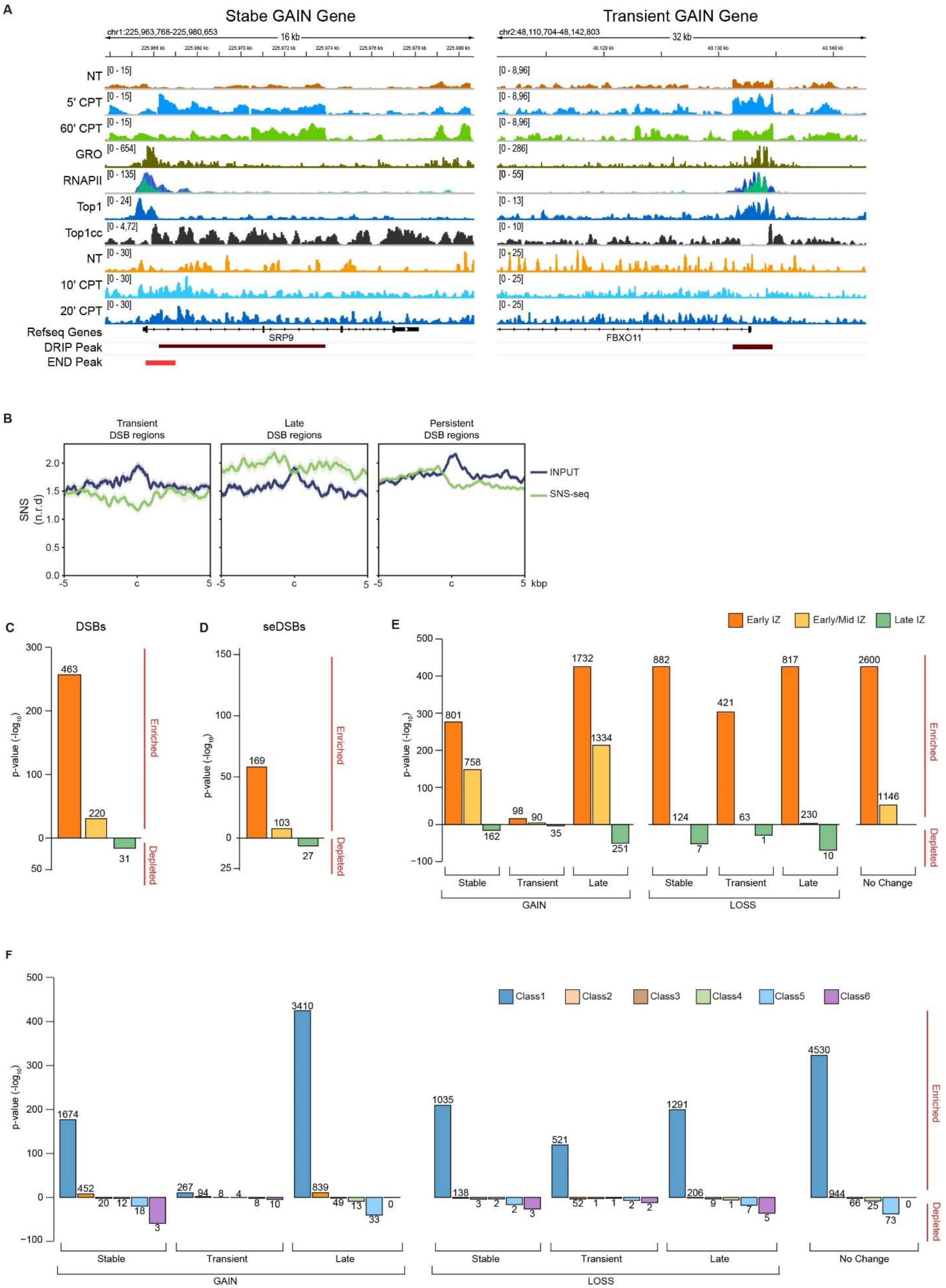

A
Stabe GAIN Gene
Transient GAIN Gene
chr1:225,963,768-225,980,653
16 kb
chr2:48,110,704-48,142,803
32 kb
NT
5' CPT
60' CPT
GRO
RNAPII
Top1
Top1cc
NT
10' CPT
20' CPT
Refseq Genes
DRIP Peak
END Peak
SRP9
FBXO11
B
Transient DSB regions
Late DSB regions
Persistent DSB regions
SNS (n.r.d)
INPUT
SNS-seq
kbp
C
DSBs
p-value (-log10)
463
220
31
Enriched
Depleted
D
seDSBs
169
103
27
E
Early IZ
Early/Mid IZ
Late IZ
801
758
162
98
90
35
1732
1334
251
882
124
7
421
63
1
817
230
10
2600
1146
Stable
Transient
Late
No Change
GAIN
LOSS
F
Class1
Class2
Class3
Class4
Class5
Class6
1674
452
20
12
18
3
267
94
8
4
8
10
3410
839
49
13
33
0
1035
138
3
2
2
3
521
52
1
1
2
2
1291
206
9
1
7
5
4530
944
66
25
73
0

Figure S6. DSB clusters and hybrids are enriched at early replication initiation zones. **A.** Integrative Genomics Viewer (IGV) screenshots of DRIP-seq (NT, 5' CPT and 10' CPT), GRO-seq, RNAPII ChIP-seq (combined tracks for NT in green and for 10'CPT in blue), Top1 ChIP-seq, Top1cc-seq and END-seq (NT, 10' CPT and 20' CPT) levels at representative genomic loci for stable and transient GAIN-associated genes. DRIP peak and END peak rows indicate the DRIP peak region the DSB cluster region, respectively. **B.** Metaplot of SNS-seq and its respective input normalized levels for Transient, Late and Persistent DSB regions. Color code as in legend. "c" indicates the middle point of the region in a window of +/- 5 kbp. SNS-seq and input signals are reported as normalized read density (n.r.d.). **C-D.** Enrichment analysis of observed Persistent DSBs (C), and seDSBs (D) at Early, Early-Mid and Late IZs *vs* expected by genome randomization (n=100). Barplots report binomial test p-values (*y*-axis) of enrichment. $-\log_{10}$(p-value) are referred as "Enriched" for a positive overlap enrichment over expected, or "Depleted" for a depletion over expected. Numbers of observed overlaps at each category are reported. **E.** Enrichment analysis of R-loop peak categories at Early, Early-Mid and Late IZs *vs* expected by genome randomization (n=100). Barplots report binomial test p-values (*y*-axis) of enrichment. $-\log_{10}$(p-value) are referred as "Enriched" for a positive overlap enrichment over expected, or "Depleted" for a depletion over expected. Numbers of observed overlaps at each category are reported. **F.** Enrichment analysis of R-loop peak categories at different TAD boundary classes *vs* expected by genome randomization (n=100). Barplots report binomial test p-values (*y*-axis) of enrichment. $-\log_{10}$(p-value) are referred as "Enriched" for a positive overlap enrichment over expected, or "Depleted" for a depletion over expected. Numbers of observed overlaps at each category are reported.

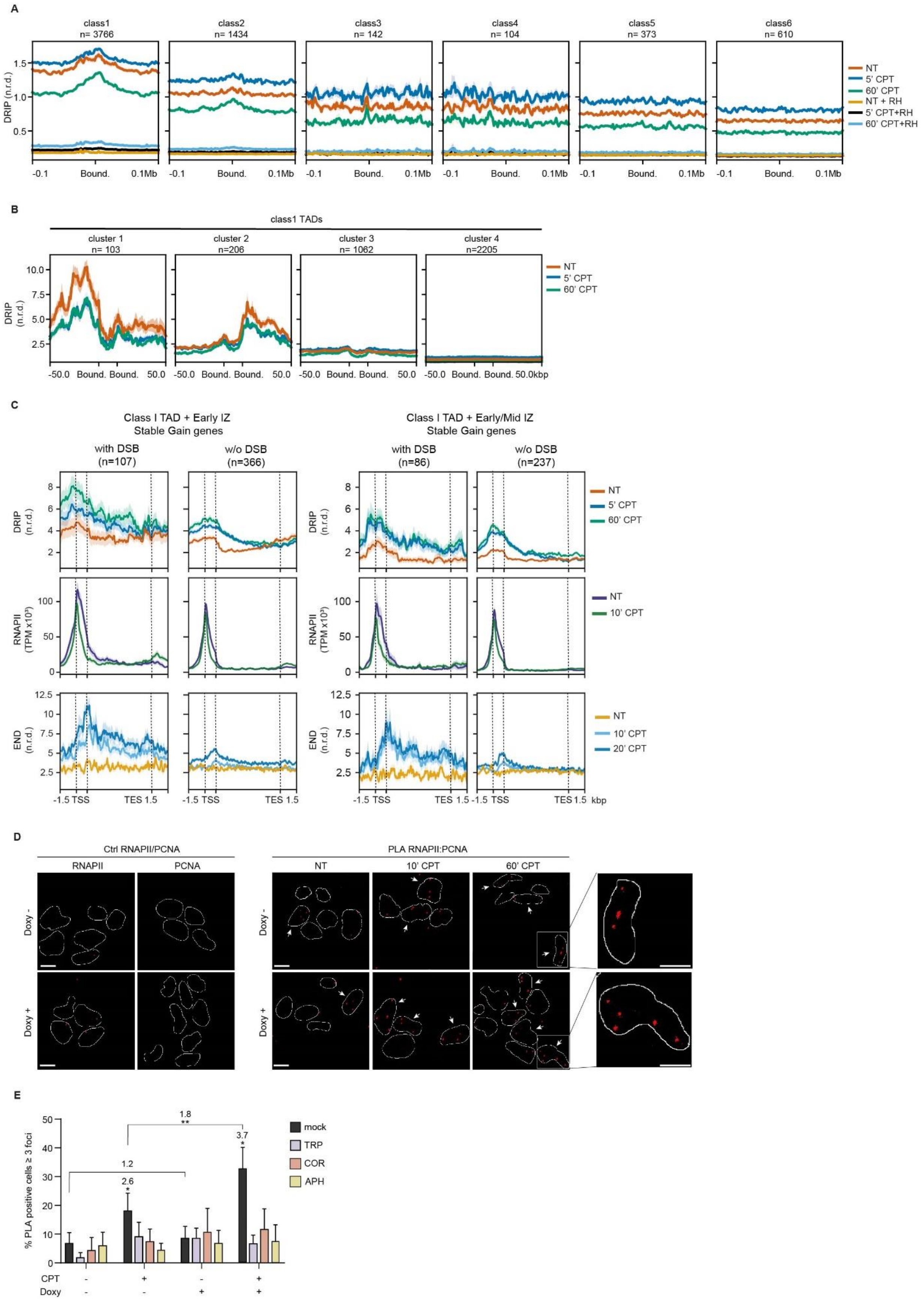

A
class1
n= 3766
class2
n= 1434
class3
n= 142
class4
n= 104
class5
n= 373
class6
n= 610
DRIP (n.r.d.)
-0.1
Bound.
0.1Mb
NT
5' CPT
60' CPT
NT + RH
5' CPT+RH
60' CPT+RH
B
class1 TADs
cluster 1
n= 103
cluster 2
n=206
cluster 3
n= 1062
cluster 4
n=2205
DRIP (n.r.d.)
-50.0 Bound. Bound. 50.0
-50.0 Bound. Bound. 50.0kbp
NT
5' CPT
60' CPT
C
Class I TAD + Early IZ
Stable Gain genes
Class I TAD + Early/Mid IZ
Stable Gain genes
with DSB
(n=107)
w/o DSB
(n=366)
with DSB
(n=86)
w/o DSB
(n=237)
DRIP (n.r.d.)
RNAPII (TPM x10³)
END (n.r.d.)
NT
5' CPT
60' CPT
NT
10' CPT
NT
10' CPT
20' CPT
-1.5 TSS TES 1.5 kbp
D
Ctrl RNAPII/PCNA
RNAPII
PCNA
PLA RNAPII:PCNA
NT
10' CPT
60' CPT
Doxy -
Doxy +
E
% PLA positive cells ≥ 3 foci
mock
TRP
COR
APH
1.8
**
3.7
*
1.2
2.6
*
CPT
Doxy

Figure S7. Hybrids are enriched at class 1 TAD boundaries. **A.** Metaplot of non-treated (NT), 5' CPT and 60' CPT DRIP-Seq signal at TAD boundaries classes. "RH" indicates samples treated with RNaseH1. Colors as in legend. "Bound." indicates the TAD boundary in a window of +/- 0.1Mb. DRIP-seq levels are reported as mean of normalized read density (n.r.d.). **B.** Metaplot of non-treated (NT), 5' CPT and 60' CPT DRIP-Seq signal normalized levels at class1 TAD boundaries in a window of +/- 50 kb. Colors as in legend. "Bound." indicate TAD Start Site and TAD End Site. Class 1 boundaries were divided into four *k*-means clusters on the base of DRIP-seq levels. This categorization allows to highlight that most of class 1 boundaries (clusters 3 and 4) have very low level of DRIP-seq, while clusters 1 and 2, that are fewer, have high level of DRIP-seq signal that is enriched asymmetrically between the two genomic regions upstream and downstream of the TAD. **C.** Metaplots of DRIP-Seq, RNAPII and END-Seq at gene regions associated to Stable Gain hybrids with Persistent DSBs at Class1 TAD and Early IZ (left) and at gene regions associated to Stable Gain hybrids without Persistent DSBs at Class1 TAD and Early IZ (right). Colors as in legend. TSS and TES indicate Transcription Start Site and Transcription End Site of genes. **D**. Representative images of RNAPII:PCNA proximity ligation assay (PLA) with relative negative control samples (Ctrl RNAPII/PCNA). **E**. PLA of Proliferating Cell Nuclear Antigen (PCNA, Ab PC11, sc-53407) with RNAPII (Ab H-224, sc-9001) in cells expressing (+ doxy) or not (- doxy) TFIISm. After TFIISm over-expression cells were treated with 1 µM triptolide (TRP), with 12.5 µg/mL cordycepin (COR) or with 1 µM aphidicolin (APH) for 2 hours prior to 10 minutes of CPT treatment. Fold change of CPT treated versus untreated samples and doxycycline-induced versus not doxycycline-induced samples is reported. Each bar represents the mean value ± SEM of three independent experiments. Average number of cells analyzed is 400. Statistical significance was calculated with one-tailed paired t-test. *p* values are: ∗, <0.05; ∗∗, <0.01, ∗∗∗, <0.001, ∗∗∗∗, <0.0001.

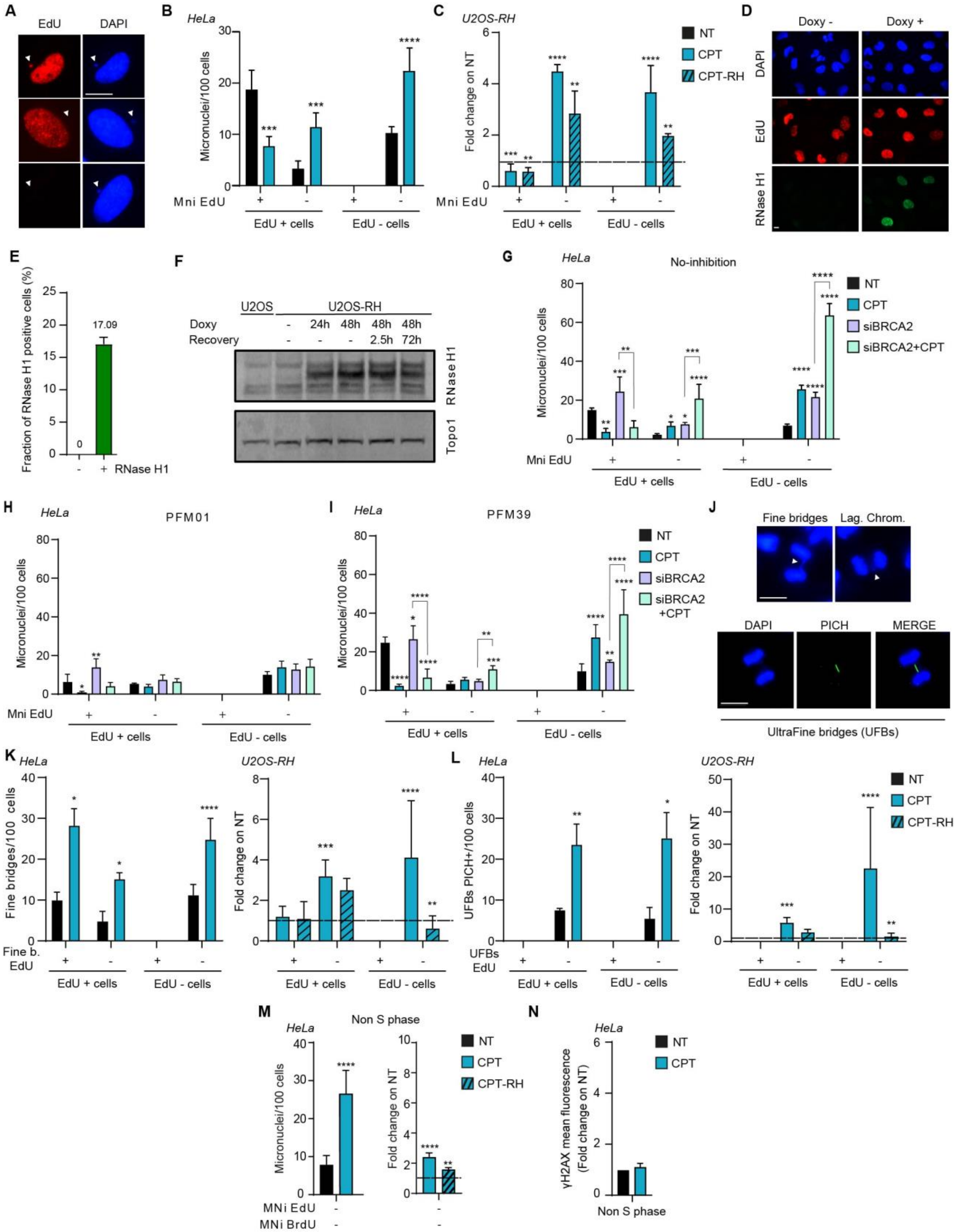

Figure S8. Top1cc induction during non-S phase can trigger mitotic errors and micronuclei mediated by DNA-RNA hybrids. A. Representative images of both EdU+/- cells and EdU+/-

micronuclei. **B.** Micronuclei increase in Hela cells after 1 hour CPT treatment in EdU labeled cells. EdU+ and EdU- micronuclei were distinguished in both EdU+ and EdU- cell populations. For each bar plot the number of micronuclei is reported as micronuclei (MNi)/100 cells. Average number of cells analyzed is 470. Each bar represents the mean value ± SEM. Statistical significance was calculated considering micronuclei distribution of treated samples compared to not-treated samples. $*p < 0.05$, $**p < 0.01$, $***p < 0.001$, $****p < 0.0001$ (two-tailed Mann-Whitney test). At least three independent experiments are reported. **C.** To evaluate the dependance of micronuclei increase on R-loops, U2OS cells were induced with doxycycline for the overexpression of RNaseH1 and treated after 48 hours with CPT for 1 hour. Micronuclei quantitation in induced versus not-induced cells is reported as fold change of micronuclei/100 cells. The average number of cells analyzed is 410. Each bar represents the mean value ± SEM. Statistical significance was calculated considering micronuclei distribution of treated samples compared to not-treated samples. $*p < 0.05$, $**p < 0.01$, $***p < 0.001$, $****p < 0.0001$ (two-tailed Mann-Whitney test). At least three independent experiments are reported. **D.** Representative images of U2OS-RH cells stained with DAPI, EdU, and anti-FLAG antibody to detect RNaseH1 expression (green fluorescence). **E.** Percentage of cells that express RNaseH1-FLAG detected by IF. **F.** Western Blot showing RNaseH1 overexpression, detected by using an anti-FLAG antibody, in U2OS-RH cells treated for 24 hours and 48 hours with doxycycline. Top1 as loading control. **G-I.** Micronuclei increase after 1 hour CPT treatment in EdU labelled HeLa cells silenced for BRCA2 repair factor and treated with MRE11 inhibitors (PFM01, PFM39). EdU+ and EdU- micronuclei were distinguished in both EdU+ and EdU- cell populations. For each bar plot the number of micronuclei is reported as micronuclei/100 cells. The average number of cells analyzed is 310 (G), 400 (H), 310 (I). Each bar represents the mean value ± SEM. Statistical significance was calculated considering micronuclei distribution of treated/inhibited samples compared to not-treated samples. $*p < 0.05$, $**p < 0.01$, $***p < 0.001$, $****p < 0.0001$, (two-tailed Mann-Whitney test). At least three independent experiments are reported. **J.** Representative images of HeLa cells stained with DAPI to detect fine bridges, lagging chromosomes and ultrafine bridges (PICH staining in green) after 1 hour of CPT treatment and mitotic shake-off after about 20 hours of recovery in fresh medium. **K-L.** Same as in (B and C) but detecting the number of fine bridges and ultrafine bridges after DAPI and EdU staining in HeLa (left panel) and U2OS-RH cells (right panel). The average number of cells analyzed is 170 (K), 230 (L). Each bar represents the mean value ± SEM. Statistical significance was calculated considering micronuclei distribution of treated samples compared to not-treated samples. $*p < 0.05$, $**p < 0.01$, $***p < 0.001$, $****p < 0.0001$ (two-tailed Mann-Whitney test). At least three independent experiments are reported. **M.** Micronuclei increase in dual labeled cells that were in non-S phase during 1 hour CPT administration. For each bar plot the number of micronuclei is reported as micronuclei/100 cells either not normalized or normalized on not treated cells. The average number of cells analyzed is 1000. Each bar represents the mean value ± SEM. Statistical significance was calculated comparing micronuclei distribution of treated over non-treated samples. $*p < 0.05$, $**p < 0.01$, $***p < 0.001$, $****p < 0.0001$ (two-tailed Mann-Whitney test). **N.** Phosphorylation of histone H2AX (γ-H2AX) detected by immunofluorescence in HeLa cells in non-S phase during 1 hour CPT treatment and dual labeled as in figure 6D. The total nuclear fluorescence is reported as fold change on not-treated cells. The average number of cells analyzed is 860. Each bar represents the mean value ± SEM. Statistical significance was

calculated considering fold change of γ-H2AX signals of treated samples *vs* untreated samples. $*p < 0.05$, $**p < 0.01$, $***p < 0.001$, $****p < 0.0001$ (one-tailed ratio paired *t* test).

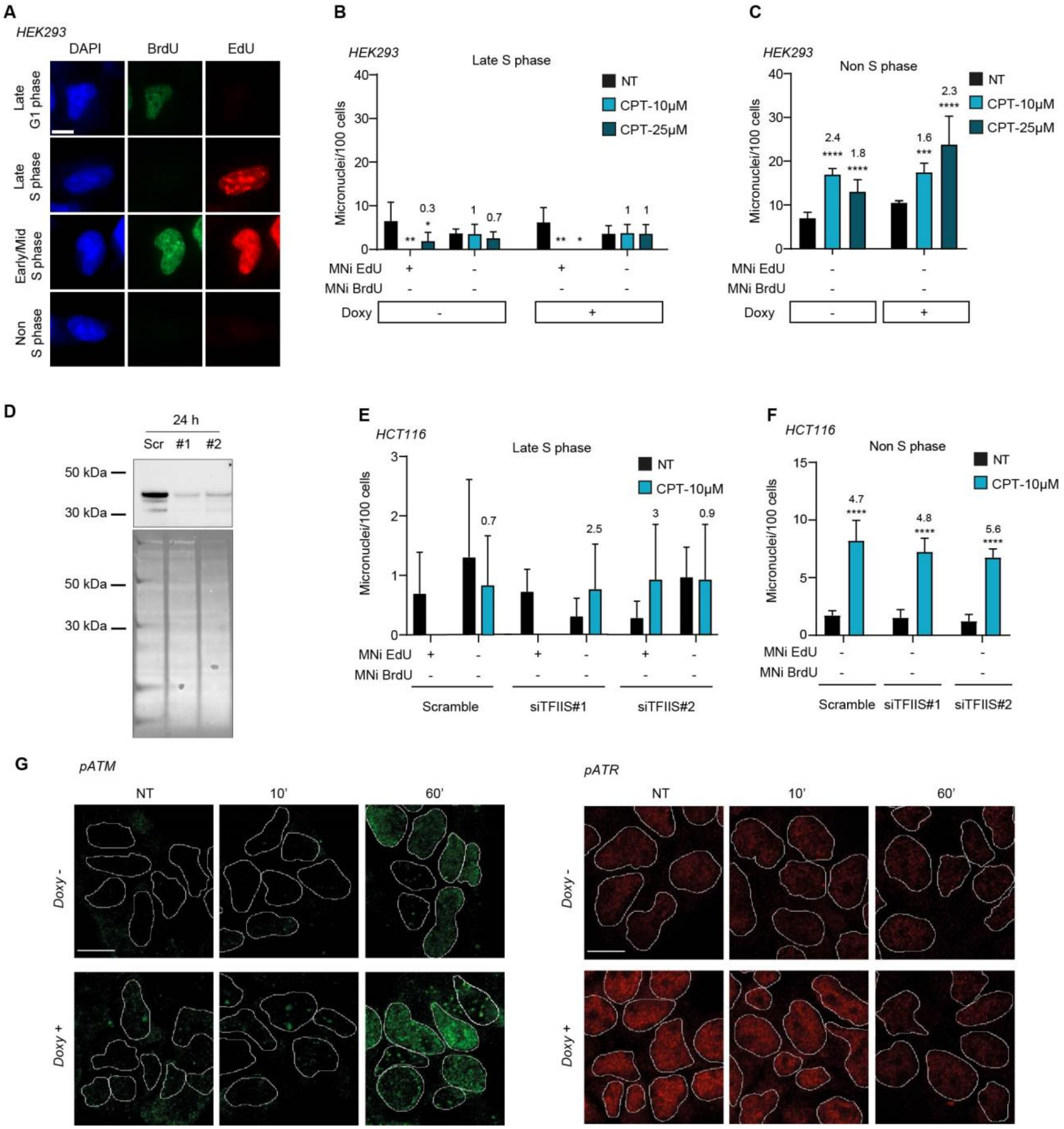

Figure S9. Elongation factors TFIISm can increase micronuclei when Top1cc induction occurs in non-S phase cells. A. Representative images of dual labelled TFIISm expressing HEK293 cells in different cell cycle phases. **B-C.** Micronuclei quantitation in HEK293 cells, overexpressing or not TFIISm, that were in late S-phase (B) or non-S phase (C) during 1h CPT administration. For each bar plot the number of micronuclei is reported as micronuclei/100 cells. Average number of cells is 120 (B) and 700 (C). Each bar represents the mean value ± SEM. Statistical significance was calculated comparing micronuclei distribution of treated over non-

treated samples. *p < 0.05, **p < 0.01, ***p < 0.001, ****p < 0.0001 (two-tailed Mann-Whitney test). Three independent experiments are reported. **D.** Silencing of TFIIS in HCT116 cells by western blot. **E-F.** Micronuclei quantitation in HCT116 cells silenced for TFIIS, with two different siRNAs (siTFIIS#1, siTFIIS#2), that were in late S-phase (E) or non-S phase (F) during 1h CPT administration. For each bar plot the number of micronuclei is reported as micronuclei/100 cells. Average number of cells is 75 (E) and 350 (F). Each bar represents the mean value ± SEM of three independent experiments. Statistical significance was calculated comparing micronuclei distribution of treated over non-treated samples. *p < 0.05, **p < 0.01, ***p < 0.001, ****p < 0.0001 (two-tailed Mann-Whitney test). In (E) panel, all the increases are not statistically significant **G.** Representative images of ATM and ATR phosphorylation (ATM Ser1981 and ATR Ser428) by immunofluorescence in HEK293m cells induced (Doxy +) or not induced (Doxy -) for TFIISm expression.

Table S2. Primer sequences.

SNRPN_Fw	TGCCAGGAAGCCAAATGAGT	(*26*)
SNRPN_Rv	TCCCTCTTGGCAACATCCA	(*26*)
EGR1_Fw	GCCAAGTCCTCCCTCTCTACTG	(*26*)
EGR1_Rv	GGAAGTGGGCAGAAAGGATTG	(*26*)
RPL13A_Fw	GCTTCCAGCACAGGACAGGTAT	(*26*)
RPL13A_Rv	CAC CCACTACCCGAGTTCAAG	(*26*)
ACTB_Fw	GGAGCTGTCACATCCAGGGTC	(*27*)
ACTB_Rv	TGCTGATCCACATCTGCTGG	(*27*)
SP2_Fw	GCCTAGTGCCTACCAAGTGC	(*28*)
SP2_Rv	CGTGTGCATCTGAATCATCC	(*28*)
CASP7_Fw	GCACAGCTAATACAGATGCTCCT	This work
CASP7_Rv	AGGCTAGGCTAAGCTCTGAAATT	This work
YWHAG_Fw	AGCTAGAGGTCACAGATTTGCAT	This work
YWHAG_Rv	GAATCGAGCACTTCATATTCCA	This work
UpDDX18_Fw	CTAGTTCTGCACCCACAGAGG	This work
UpDDX18_Rv	TGGCTTCACACTGTGCTAAAG	This work
NONO_FW	TCATTTGCCATGAAACTGCTAC	This work
NONO_Rv	ATCCTGTGACTCTTGCCACATA	This work
ELOC_Fw	AAAGAGGTGAGAGTTTGGGAAGT	This work
ELOC_Rv	CATGGCTTGGTCAGTATCTCAGT	This work
CD1_Fw	GGAGTTAAACAGGAGCAGGAGA	This work
CD1_Rv	GTCATTGTCGTCATCGTTGC	This work
RNF43_Fw	AGTTATTTCAGCAACACCAGCA	This work
RNF43_Rv	GAAAGATCAGCAGAACAGAAAGC	This work
FAM177A1_Fw	GGGTTCTTCTTTCGAGGGTATT	This work
FAM177A1_Rv	AAAGGCTAGTGATGGGATGTG	This work
SF3B3_Fw	CATTTCCTGTGGGTTATTAGGG	This work
SF3B3_Rv	GGAATCTCGTTTCTGATTGCTT	This work
SMIM41_Fw	AGGCTTTCTTCATCCAACTTCC	This work
SMIM41_Rv	TTCCACAGCAAACATCTCAGTC	This work
GPS1_Fw	AAGTCTCTGTTGATGGGCAAG	This work
GPS1_Rv	CACCTTGACATGGATCTGGTT	This work
UpKLHDC8B_Fw	AGGTCGGACGCTAAATTCTGT	This work
UpKLHDC8B_Rv	AAACCTCCTCTCGCCTTAAC	This work
SOX7_Fw	CTGAATCCAAGCTATTGCCTCT	This work
SOX7_Rv	CTCTCGGTTCCCTTCAATAACA	This work
RF_Fw	ACTCAGCACAACCAAGGATCA	This work

RF_Rv	ATTCGGAGGGTTTAGAGGGTTC	This work
PDC1_Fw	GAAGGTATGAGATGGGCTGGTAA	(87)
PDC1_Rv	CCTTGATACGAGCGTAACCATCA	(87)
DEK_Fw	TAGCGATGAAGAGAGCGAGA	This work
DEK_Rv	GCTCAGGTTTCTTGGGCTTAT	This work
DPM1_Fw	GCGTAAGAAAGGGATCACGA	This work
DPM1_Rv	AAGAATGAGATGAGCTGGCTTC	This work
ATP5MC2_Fw	AGGGATTGGTGACTTTAGACGA	This work
ATP5MC2_Rv	CATCCTGACTTGTGCCTTCTTT	This work
UBE2G2_Fw	CTTCATTAGAGGGAAAGCCAAA	This work
UBE2G2_Rv	AGTGAGAGCTTGAGGAAGAAGG	This work
TMEM209_Fw	GAAGGGCACGTACTTATGATGG	This work
TMEM209_Rv	TGAGCCTAGCTGAAGAAAGGAG	This work
RFT1_Fw	GCCACCTGATCTAATTCACTCC	This work
RFT1_Rv	GAGATAGGAGCCTGCCTTTAGA	This work
CLTA_Fw	CGAGAGGACTTGTCTGGAAACT	This work
CLTA_Rv	TGCTCCCTAACCTCCTAATTCA	This work
NIN_Fw	CTGGAGAAACGGGACAATACTT	This work
NIN_Rv	ATATGGAAATGACGCTCACG	This work
AP1G1_Fw	AAGACACAAGAACGCTGATACG	This work
AP1G1_Rv	TCCTTGAACCCTTGACTTTCTC	This work
PTCD1_Fw	TGCATCGAACCTTACATGGTAT	This work
PTCD1_Rv	CCAGTTGCTTTGCACTTGAAT	This work
AREL1_Fw	AAACAAGGAAGTATGCGACCA	This work
AREL1_Rv	TTAGAATATCCCTGGGCAGTTG	This work
DACH2_Fw	TACACCAAGCTGAAGAGACTGG	This work
DACH2_Rv	GATGAGTTTGCAGCGGTTTAC	This work
α-SAT_Fw	AGACACAAGCATTCTCAGCAA	(*28*)
α-SAT_Rv	CTTTTTCATCATAGGCCTCAA	(*28*)

Other Supplementary Materials

Table S1. DRIP-seq and END-seq sequencing reports and alignment statistics.
Excel file containing FastQC sequencing report (N. of sequenced reads and % of duplicated reads) and alignment report (N. of mapped and filtered reads) for DRIP-seq and END-seq libraries.

REFERENCES AND NOTES

1. Y. Pommier, Y. Sun, S.-Y. N. Huang, J. L. Nitiss, Roles of eukaryotic topoisomerases in transcription, replication and genomic stability. *Nat. Rev. Mol. Cell Biol.* **17**, 703–721 (2016).

2. G. Capranico, J. Marinello, G. Chillemi, Type I DNA topoisomerases. *J. Med. Chem.* **60**, 2169–2192 (2017).

3. Y. Pommier, Topoisomerase I inhibitors: camptothecins and beyond. *Nat. Rev. Cancer* **6**, 789–802 (2006).

4. Y. Pommier, A. Nussenzweig, S. Takeda, C. Austin, Human topoisomerases and their roles in genome stability and organization. *Nat. Rev. Mol. Cell Biol.* **23**, 407–427 (2022).

5. J. Marinello, G. Chillemi, S. Bueno, S. G. Manzo, G. Capranico, Antisense transcripts enhanced by camptothecin at divergent CpG-island promoters associated with bursts of topoisomerase I-DNA cleavage complex and R-loop formation. *Nucleic Acids Res.* **41**, 10110–10123 (2013).

6. L. Baranello, D. Wojtowicz, K. Cui, B. N. Devaiah, H. J. Chung, K. Y. Chan-Salis, R. Guha, K. Wilson, X. Zhang, H. Zhang, J. Piotrowski, C. J. Thomas, D. S. Singer, B. F. Pugh, Y. Pommier, T. M. Przytycka, F. Kouzine, B. A. Lewis, K. Zhao, D. Levens, RNA polymerase II regulates topoisomerase 1 activity to favor efficient transcription. *Cell* **165**, 357–371 (2016).

7. L. Baranello, D. Bertozzi, M. V. Fogli, Y. Pommier, G. Capranico, DNA topoisomerase I inhibition by camptothecin induces escape of RNA polymerase II from promoter-proximal pause site, antisense transcription and histone acetylation at the human HIF-1alpha gene locus. *Nucleic Acids Res.* **38**, 159–171 (2010).

8. A. Khobta, F. Ferri, L. Lotito, A. Montecucco, R. Rossi, G. Capranico, Early effects of topoisomerase I inhibition on RNA polymerase II along transcribed genes in human cells. *J. Mol. Biol.* **357**, 127–138 (2006).

9. P. Duann, M. Sun, C. T. Lin, H. Zhang, L. F. Liu, Plasmid linking number change induced by topoisomerase I-mediated DNA damage. *Nucleic Acids Res.* **27**, 2905–2911 (1999).

10. F. Kouzine, A. Gupta, L. Baranello, D. Wojtowicz, K. Ben-Aissa, J. Liu, T. M. Przytycka, D. Levens, Transcription-dependent dynamic supercoiling is a short-range genomic force. *Nat. Struct. Mol. Biol.* **20**, 396–403 (2013).

11. A. A. Cohen, N. Geva-Zatorsky, E. Eden, M. Frenkel-Morgenstern, I. Issaeva, A. Sigal, R. Milo, C. Cohen-Saidon, Y. Liron, Z. Kam, L. Cohen, T. Danon, N. Perzov, U. Alon, Dynamic proteomics of individual cancer cells in response to a drug. *Science* **322**, 1511–1516 (2008).

12. S. D. Desai, T.-K. Li, A. Rodriguez-Bauman, E. H. Rubin, L. F. Liu, Ubiquitin/26S proteasome-mediated degradation of topoisomerase I as a resistance mechanism to camptothecin in tumor cells. *Cancer Res.* **61**, 5926–5932 (2001).

13. B. Gómez-González, A. Aguilera, Transcription-mediated replication hindrance: A major driver of genome instability. *Genes Dev.* **33**, 1008–1026 (2019).

14. M. P. Crossley, M. Bocek, K. A. Cimprich, R-loops as cellular regulators and genomic threats. *Mol. Cell* **73**, 398–411 (2019).

15. T. García-Muse, A. Aguilera, R loops: From physiological to pathological roles. *Cell* **179**, 604–618 (2019).

16. S. Hamperl, M. J. Bocek, J. C. Saldivar, T. Swigut, K. A. Cimprich, Transcription-replication conflict orientation modulates r-loop levels and activates distinct DNA damage responses. *Cell* **170**, 774–786.e19 (2017).

17. M. Holmström, V. Winters, Micronucleus induction by camptothecin and amsacrine in bone marrow of male and female CD-1 mice. *Mutagenesis* **7**, 189–193 (1992).

18. J. Marinello, A. Arleo, M. Russo, M. Delcuratolo, F. Ciccarelli, Y. Pommier, G. Capranico, Topoisomerase I poison-triggered immune gene activation is markedly reduced in human

small-cell lung cancers by impairment of the cGAS/STING pathway. *Br. J. Cancer* **127**, 1214–1225 (2022).

19. Y. H. Hsiang, M. G. Lihou, L. F. Liu, Arrest of replication forks by drug-stabilized topoisomerase I-DNA cleavable complexes as a mechanism of cell killing by camptothecin. *Cancer Res.* **49**, 5077–5082 (1989).

20. D. Strumberg, A. A. Pilon, M. Smith, R. Hickey, L. Malkas, Y. Pommier, Conversion of topoisomerase i cleavage complexes on the leading strand of ribosomal DNA into 5′-phosphorylated DNA double-strand breaks by replication runoff. *Mol. Cell. Biol.* **20**, 3977–3987 (2000).

21. M. Regairaz, Y.-W. Zhang, H. Fu, K. K. Agama, N. Tata, S. Agrawal, M. I. Aladjem, Y. Pommier, Mus81-mediated DNA cleavage resolves replication forks stalled by topoisomerase I-DNA complexes. *J. Cell Biol.* **195**, 739–749 (2011).

22. H. Fu, M. M. Martin, M. Regairaz, L. Huang, Y. You, C.-M. Lin, M. Ryan, R. Kim, T. Shimura, Y. Pommier, M. I. Aladjem, The DNA repair endonuclease Mus81 facilitates fast DNA replication in the absence of exogenous damage. *Nat. Commun.* **6**, 6746 (2015).

23. S. Di Marco, Z. Hasanova, R. Kanagaraj, N. Chappidi, V. Altmannova, S. Menon, H. Sedlackova, J. Langhoff, K. Surendranath, D. Hühn, R. Bhowmick, V. Marini, S. Ferrari, I. D. Hickson, L. Krejci, P. Janscak, RECQ5 helicase cooperates with mus81 endonuclease in processing stalled replication forks at common fragile sites during mitosis. *Mol. Cell.* **66**, 658–671.e8 (2017).

24. B. Pardo, M. Moriel-Carretero, T. Vicat, A. Aguilera, P. Pasero, Homologous recombination and Mus81 promote replication completion in response to replication fork blockage. *EMBO Rep.* **21**, e49367 (2020).

25. N. Chappidi, Z. Nascakova, B. Boleslavska, R. Zellweger, E. Isik, M. Andrs, S. Menon, J. Dobrovolna, C. Balbo Pogliano, J. Matos, A. Porro, M. Lopes, P. Janscak, Fork cleavage-

religation cycle and active transcription mediate replication restart after fork stalling at Co-transcriptional R-loops. *Mol. Cell* **77**, 528–541.e8 (2020).

26. P. Pourquier, L. M. Ueng, J. Fertala, D. Wang, H. J. Park, J. M. Essigmann, M. A. Bjornsti, Y. Pommier, Induction of reversible complexes between eukaryotic DNA topoisomerase I and DNA-containing oxidative base damages. 7, 8-dihydro-8-oxoguanine and 5-hydroxycytosine. *J. Biol. Chem.* **274**, 8516–8523 (1999).

27. S. Katyal, Y. Lee, K. C. Nitiss, S. M. Downing, Y. Li, M. Shimada, J. Zhao, H. R. Russell, J. H. J. Petrini, J. L. Nitiss, P. J. McKinnon, Aberrant topoisomerase-1 DNA lesions are pathogenic in neurodegenerative genome instability syndromes. *Nat. Neurosci.* **17**, 813–821 (2014).

28. H. Takashima, C. F. Boerkoel, J. John, G. M. Saifi, M. A. M. M. Salih, D. Armstrong, Y. Mao, F. A. Quiocho, B. B. Roa, M. Nakagawa, D. W. Stockton, J. R. Lupski, Mutation of TDP1, encoding a topoisomerase I-dependent DNA damage repair enzyme, in spinocerebellar ataxia with axonal neuropathy. *Nat. Genet.* **32**, 267–272 (2002).

29. R. Hirano, H. Interthal, C. Huang, T. Nakamura, K. Deguchi, K. Choi, M. B. Bhattacharjee, K. Arimura, F. Umehara, S. Izumo, J. L. Northrop, M. A. M. Salih, K. Inoue, D. L. Armstrong, J. J. Champoux, H. Takashima, C. F. Boerkoel, Spinocerebellar ataxia with axonal neuropathy: Consequence of a Tdp1 recessive neomorphic mutation? *EMBO J.* **26**, 4732–4743 (2007).

30. N. Kim, S. N. Huang, J. S. Williams, Y. C. Li, A. B. Clark, J.-E. Cho, T. A. Kunkel, Y. Pommier, S. Jinks-Robertson, Mutagenic processing of ribonucleotides in DNA by yeast topoisomerase I. *Science* **332**, 1561–1564 (2011).

31. S. M. Cerritelli, J. Iranzo, S. Sharma, A. Chabes, R. J. Crouch, D. Tollervey, A. El Hage, High density of unrepaired genomic ribonucleotides leads to topoisomerase 1-mediated severe growth defects in absence of ribonucleotide reductase. *Nucleic Acids Res.* **48**, 4274–4297 (2020).

32. A. Aguilera, B. Gómez-gonzález, DNA-RNA hybrids : The risks of DNA breakage during transcription. *Nat. Struct. Mol. Biol.* **24**, 439–443 (2017).

33. J. Marinello, S. Bertoncini, I. Aloisi, A. Cristini, G. M. Tagliazucchi, M. Forcato, O. Sordet, G. Capranico, M. M. Tagliazucchi, G. Forcato, Dynamic effects of topoisomerase I inhibition on R-loops and short transcripts at active promoters. *PLOS ONE* **11**, e0147053 (2016).

34. A. Cristini, G. Ricci, S. Britton, S. Salimbeni, S. Huang, J. Marinello, P. Calsou, Y. Pommier, G. Favre, G. Capranico, N. Gromak, O. Sordet, Dual processing of R-loops and topoisomerase I induces transcription-dependent DNA double-strand breaks. *Cell Rep.* **28**, 3167–3181.e6 (2019).

35. K. Bartsch, K. Knittler, C. Borowski, S. Rudnik, M. Damme, K. Aden, M. E. Spehlmann, N. Frey, P. Saftig, A. Chalaris, B. Rabe, Absence of RNase H2 triggers generation of immunogenic micronuclei removed by autophagy. *Hum. Mol. Genet.* **26**, 3960–3972 (2017).

36. A. M. S. Giordano, M. Luciani, F. Gatto, M. A. Alezz, C. Beghè, L. Della Volpe, A. Migliara, S. Valsoni, M. Genua, M. Dzieciatkowska, G. Frati, J. Tahraoui-Bories, S. C. Giliani, S. Orcesi, E. Fazzi, R. Ostuni, A. D'Alessandro, R. Di Micco, I. Merelli, A. Lombardo, M. A. M. Reijns, N. Gromak, A. Gritti, A. Kajaste-Rudnitski, DNA damage contributes to neurotoxic inflammation in Aicardi-Goutières syndrome astrocytes. *J. Exp. Med.* **219**, e20211121 (2022).

37. A. Cristini, M. Tellier, F. Constantinescu, C. Accalai, L. O. Albulescu, R. Heiringhoff, N. Bery, O. Sordet, S. Murphy, N. Gromak, RNase H2, mutated in Aicardi-Goutières syndrome, resolves co-transcriptional R-loops to prevent DNA breaks and inflammation. *Nat. Commun.* **13**, 2961 (2022).

38. Z. Andrysik, M. D. Galbraith, A. L. Guarnieri, S. Zaccara, K. D. Sullivan, A. Pandey, M. MacBeth, A. Inga, J. M. Espinosa, Identification of a core TP53 transcriptional program with highly distributed tumor suppressive activity. *Genome Res.* **27**, 1645–1657 (2017).

39. Y. Luo, B. C. Hitz, I. Gabdank, J. A. Hilton, M. S. Kagda, B. Lam, Z. Myers, P. Sud, J. Jou, K. Lin, U. K. Baymuradov, K. Graham, C. Litton, S. R. Miyasato, J. S. Strattan, O. Jolanki, J. W. Lee, F. Y. Tanaka, P. Adenekan, E. O'Neill, J. M. Cherry, New developments on the encyclopedia of DNA elements (ENCODE) data portal. *Nucleic Acids Res.* **48**, D882–D889 (2020).

40. L. Lotito, A. Russo, G. Chillemi, S. Bueno, D. Cavalieri, G. Capranico, Global transcription regulation by DNA topoisomerase i in exponentially growing *Saccharomyces cerevisiae* cells: Activation of telomere-proximal genes by TOP1 deletion. *J. Mol. Biol.* **377**, 311–322 (2008).

41. J. Marinello, G. Capranico, in *Methods in Molecular Biology* (Humana Press Inc., 2022), vol. 2528, pp. 203–213.

42. D. Roy, Z. Zhang, Z. Lu, C.-L. Hsieh, M. R. Lieber, Competition between the RNA transcript and the nontemplate DNA strand during R-loop formation in vitro: A nick can serve as a strong R-loop initiation site. *Mol. Cell. Biol.* **30**, 146–159 (2010).

43. L. Chen, J.-Y. Chen, X. Zhang, Y. Gu, R. Xiao, C. Shao, P. Tang, H. Qian, D. Luo, H. Li, Y. Zhou, D.-E. Zhang, X.-D. Fu, R-ChIP using inactive RNase H reveals dynamic coupling of R-loops with transcriptional pausing at gene promoters. *Mol. Cell* **68**, 745–757.e5 (2017).

44. S. K. Das, V. Kuzin, D. P. Cameron, S. Sanford, R. K. Jha, Z. Nie, M. T. Rosello, R. Holewinski, T. Andresson, J. Wisniewski, T. Natsume, D. H. Price, B. A. Lewis, F. Kouzine, D. Levens, L. Baranello, MYC assembles and stimulates topoisomerases 1 and 2 in a "topoisome" *Mol. Cell* **82**, 140–158.e12 (2022).

45. D. Castillo-Guzman, F. Chédin, Defining R-loop classes and their contributions to genome instability. *DNA Repair* **106**, 103182 (2021).

46. D. Zatreanu, Z. Han, R. Mitter, E. Tumini, H. Williams, L. Gregersen, A. B. Dirac-Svejstrup, S. Roma, A. Stewart, A. Aguilera, J. Q. Svejstrup, Elongation factor TFIIS prevents transcription stress and R-loop accumulation to maintain genome stability. *Mol. Cell* **76**, 57–69.e9 (2019).

47. R. M. Sheridan, N. Fong, A. D'Alessandro, D. L. Bently, Widespread backtracking by RNA Pol II Is a major effector of gene activation, 5′ pause release, termination, and transcription elongation rate. *Mol. Cell* **73**, 107–118.e4 (2019).

48. A. Canela, S. Sridharan, N. Sciascia, A. Tubbs, P. Meltzer, B. P. Sleckman, A. Nussenzweig, DNA breaks and end resection measured genome-wide by end sequencing. *Mol. Cell* **63**, 898–911 (2016).

49. L. Halász, Z. Karányi, B. Boros-Oláh, T. Kuik-Rózsa, É. Sipos, É. Nagy, Á. Mosolygó-L, A. Mázló, É. Rajnavölgyi, G. Halmos, L. Székvölgyi, RNA-DNA hybrid (R-loop) immunoprecipitation mapping: An analytical workflow to evaluate inherent biases. *Genome Res.* **27**, 1063–1073 (2017).

50. Y. Hu, B. Stillman, Origins of DNA replication in eukaryotes. *Mol. Cell* **83**, 352–372 (2023).

51. D. J. Emerson, P. A. Zhao, A. L. Cook, R. J. Barnett, K. N. Klein, D. Saulebekova, C. Ge, L. Zhou, Z. Simandi, M. K. Minsk, K. R. Titus, W. Wang, W. Gong, D. Zhang, L. Yang, S. V. Venev, J. H. Gibcus, H. Yang, T. Sasaki, M. T. Kanemaki, F. Yue, J. Dekker, C. L. Chen, D. M. Gilbert, J. E. Phillips-Cremins, Cohesin-mediated loop anchors confine the locations of human replication origins. *Nature* **606**, 812–819 (2022).

52. M. M. Martin, M. Ryan, R. G. Kim, A. L. Zakas, H. Fu, C. M. Lin, W. C. Reinhold, S. R. Davis, S. Bilke, H. Liu, J. H. Doroshow, M. A. Reimers, M. S. Valenzuela, Y. Pommier, P. S. Meltzer, M. I. Aladjem, Genome-wide depletion of replication initiation events in highly transcribed regions. *Genome Res.* **21**, 1822–1832 (2011).

53. T. Sasaki, S. Ramanathan, Y. Okuno, C. Kumagai, S. S. Shaikh, D. M. Gilbert, The Chinese hamster dihydrofolate reductase replication origin decision point follows activation of transcription and suppresses initiation of replication within transcription units. *Mol. Cell. Biol.* **26**, 1051–1062 (2006).

54. O. Brison, S. El-Hilali, D. Azar, S. Koundrioukoff, M. Schmidt, V. Nähse, Y. Jaszczyszyn, A. M. Lachages, B. Dutrillaux, C. Thermes, M. Debatisse, C.-L. Chen, Transcription-

mediated organization of the replication initiation program across large genes sets common fragile sites genome-wide. *Nat. Commun.* **10**, 5693 (2019).

55. M. J. Scherr, S. A. Wahab, D. Remus, K. E. Duderstadt, Mobile origin-licensing factors confer resistance to conflicts with RNA polymerase. *Cell Rep.* **38**, 110531 (2022).

56. B. D. Pope, T. Ryba, V. Dileep, F. Yue, W. Wu, O. Denas, D. L. Vera, Y. Wang, R. S. Hansen, T. K. Canfield, R. E. Thurman, Y. Cheng, G. Gülsoy, J. H. Dennis, M. P. Snyder, J. A. Stamatoyannopoulos, J. Taylor, R. C. Hardison, T. Kahveci, B. Ren, D. M. Gilbert, Topologically associating domains are stable units of replication-timing regulation. *Nature* **515**, 402–405 (2014).

57. G. Miglietta, M. Russo, G. Capranico, G-quadruplex–R-loop interactions and the mechanism of anticancer G-quadruplex binders. *Nucleic Acids Res.* **48**, 11942–11957 (2020).

58. R. C. Duardo, F. Guerra, S. Pepe, G. Capranico, Non-B DNA structures as a booster of genome instability. *Biochimie* **214**, 176–192 (2023).

59. D. A. Koster, K. Palle, E. S. M. Bot, M. A. Bjornsti, N. H. Dekker, Antitumour drugs impede DNA uncoiling by topoisomerase I. *Nature* **448**, 213–217 (2007).

60. S. G. Manzo, S. R. Hartono, L. A. Sanz, J. Marinello, S. De Biasi, A. Cossarizza, G. Capranico, F. Chedin, DNA topoisomerase I differentially modulates R-loops across the human genome. *Genome Biol.* **19**, 100 (2018).

61. T. García-Muse, A. Aguilera, Transcription–replication conflicts: How they occur and how they are resolved. *Nat. Rev. Mol. Cell Biol.* **17**, 553–563 (2016).

62. D. Dutta, K. Shatalin, V. Epshtein, M. E. Gottesman, E. Nudler, Linking RNA polymerase backtracking to genome instability in E. coli. *Cell* **146**, 533–43 (2011).

63. E. Nudler, RNA polymerase backtracking in gene regulation and genome instability. *Cell* **149**, 1438–1445 (2012).

64. J. Brochu, É. Vlachos-Breton, D. Irsenco, M. Drolet, Characterization of a pathway of genomic instability induced by R-loops and its regulation by topoisomerases in *E. coli*. *PLOS Genet.* **19**, e1010754 (2023).

65. G. Abdurashidova, S. Radulescu, O. Sandoval, S. Zahariev, M. B. Danailov, A. Demidovich, L. Santamaria, G. Biamonti, S. Riva, A. Falaschi, Functional interactions of DNA topoisomerases with a human replication origin. *EMBO J.* **26**, 998–1009 (2007).

66. H. Fu, C. E. Redon, B. L. Thakur, K. Utani, R. Sebastian, S.-M. M. Jang, J. M. Gross, S. Mosavarpour, A. B. Marks, S. Z. Zhuang, S. B. Lazar, M. Rao, S. T. Mencer, A. M. Baris, L. S. Pongor, M. I. Aladjem, Dynamics of replication origin over-activation. *Nat. Commun.* **12**, 3448 (2021).

67. B. L. Thakur, A. Ray, C. E. Redon, M. I. Aladjem, Preventing excess replication origin activation to ensure genome stability. *Trends Genet.* **38**, 169–181 (2022).

68. Y.-W. Zhang, M. Regairaz, J. A. Seiler, K. K. Agama, J. H. Doroshow, Y. Pommier, Poly(ADP-ribose) polymerase and XPF-ERCC1 participate in distinct pathways for the repair of topoisomerase I-induced DNA damage in mammalian cells. *Nucleic Acids Res.* **39**, 3607–3620 (2011).

69. A. De Magis, S. G. Manzo, M. Russo, J. Marinello, R. Morigi, O. Sordet, G. Capranico, DNA damage and genome instability by G-quadruplex ligands are mediated by R loops in human cancer cells. *Proc. Natl. Acad. Sci. U.S.A.* **116**, 816–825 (2019).

70. M. García-Rubio, S. I. Barroso, A. Aguilera, in *Methods in Molecular Biology* (Humana Press Inc., 2018), vol. 1672, pp. 347–361.

71. N. Wong, S. John, A. Nussenzweig, A. Canela, in *Methods in Molecular Biology* (Humana Press Inc., 2021), vol. 2153, pp. 9–31.

72. A. Collins, P. Møller, G. Gajski, S. Vodenková, A. Abdulwahed, D. Anderson, E. E. Bankoglu, S. Bonassi, E. Boutet-Robinet, G. Brunborg, C. Chao, M. S. Cooke, C. Costa, S. Costa, A. Dhawan, J. de Lapuente, C. Del Bo', J. Dubus, M. Dusinska, S. J. Duthie, N. E.

Yamani, B. Engelward, I. Gaivão, L. Giovannelli, R. Godschalk, S. Guilherme, K. B. Gutzkow, K. Habas, A. Hernández, O. Herrero, M. Isidori, A. N. Jha, S. Knasmüller, I. M. Kooter, G. Koppen, M. Kruszewski, C. Ladeira, B. Laffon, M. Larramendy, L. L. Hégarat, A. Lewies, A. Lewinska, G. E. Liwszyc, A. López de Cerain, M. Manjanatha, R. Marcos, M. Milić, V. Moraes de Andrade, M. Moretti, D. Muruzabal, M. Novak, R. Oliveira, A.-K. Olsen, N. Owiti, M. Pacheco, A. K. Pandey, S. Pfuhler, B. Pourrut, K. Reisinger, E. Rojas, E. Rundén-Pran, J. Sanz-Serrano, S. Shaposhnikov, V. Sipinen, K. Smeets, H. Stopper, J. P. Teixeira, V. Valdiglesias, M. Valverde, F. van Acker, F.-J. van Schooten, M. Vasquez, J. F. Wentzel, M. Wnuk, A. Wouters, B. Žegura, T. Zikmund, S. A. S. Langie, A. Azqueta, Measuring DNA modifications with the comet assay: A compendium of protocols. *Nat. Protoc.* **18**, 929–989 (2023).

73. M. Martin, Cutadapt removes adapter sequences from high-throughput sequencing reads. *EMBnet* **17**, 10–12 (2011).

74. H. Li, R. Durbin, Fast and accurate short read alignment with Burrows-Wheeler transform. *Bioinformatics* **25**, 1754–1760 (2009).

75. S. G. Landt, G. K. Marinov, A. Kundaje, P. Kheradpour, F. Pauli, S. Batzoglou, B. E. Bernstein, P. Bickel, J. B. Brown, P. Cayting, Y. Chen, G. DeSalvo, C. Epstein, K. I. Fisher-Aylor, G. Euskirchen, M. Gerstein, J. Gertz, A. J. Hartemink, M. M. Hoffman, V. R. Iyer, Y. L. Jung, S. Karmakar, M. Kellis, P. V. Kharchenko, Q. Li, T. Liu, X. S. Liu, L. Ma, A. Milosavljevic, R. M. Myers, P. J. Park, M. J. Pazin, M. D. Perry, D. Raha, T. E. Reddy, J. Rozowsky, N. Shoresh, A. Sidow, M. Slattery, J. A. Stamatoyannopoulos, M. Y. Tolstorukov, K. P. White, S. Xi, P. J. Farnham, J. D. Lieb, B. J. Wold, M. Snyder, ChIP-seq guidelines and practices of the ENCODE and modENCODE consortia. *Genome Res.* **22**, 1813–1831 (2012).

76. H. Li, B. Handsaker, A. Wysoker, T. Fennell, J. Ruan, N. Homer, G. Marth, G. Abecasis, R. Durbin; 1000 Genome Project Data Processing Subgroup, The sequence alignment/map format and SAMtools. *Bioinformatics* **25**, 2078–2079 (2009).

77. Y. Zhang, T. Liu, C. A. Meyer, J. Eeckhoute, D. S. Johnson, B. E. Bernstein, C. Nussbaum, R. M. Myers, M. Brown, W. Li, X. S. Liu, Model-based analysis of ChIP-seq (MACS). *Genome Biol.* **9**, R137 (2008).

78. A. R. Quinlan, I. M. Hall, BEDTools: A flexible suite of utilities for comparing genomic features. *Bioinformatics* **26**, 841–842 (2010).

79. M. E. Ritchie, B. Phipson, D. Wu, Y. Hu, C. W. Law, W. Shi, G. K. Smyth, Limma powers differential expression analyses for RNA-sequencing and microarray studies. *Nucleic Acids Res.* **43**, e47 (2015).

80. M. Russo, B. De Lucca, T. Flati, S. Gioiosa, G. Chillemi, G. Capranico, DROPA: DRIP-seq optimized peak annotator. *BMC Bioinformatics.* **20**, 414 (2019).

81. M. Lawrence, R. Gentleman, V. Carey, rtracklayer: An R package for interfacing with genome browsers. *Bioinformatics* **25**, 1841–1842 (2009).

82. F. Ramírez, D. P. Ryan, B. Grüning, V. Bhardwaj, F. Kilpert, A. S. Richter, S. Heyne, F. Dündar, T. Manke, deepTools2: A next generation web server for deep-sequencing data analysis. *Nucleic Acids Res.* **44**, W160–W165 (2016).

83. P. Danecek, J. K. Bonfield, J. Liddle, J. Marshall, V. Ohan, M. O. Pollard, A. Whitwham, T. Keane, S. A. McCarthy, R. M. Davies, Twelve years of SAMtools and BCFtools. *Gigascience* **10**, giab008 (2021).

84. A. Canela, Y. Maman, S. N. Huang, G. Wutz, W. Tang, G. Zagnoli-Vieira, E. Callen, N. Wong, A. Day, J.-M. Peters, K. W. Caldecott, Y. Pommier, A. Nussenzweig, Topoisomerase II-Induced chromosome breakage and translocation is determined by chromosome architecture and transcriptional activity. *Mol. Cell* **75**, 252–266.e8 (2019).

85. The ENCODE Project Consortium, An integrated encyclopedia of DNA elements in the human genome. *Nature* **489**, 57–74 (2012).

86. J. T. Robinson, H. Thorvaldsdóttir, W. Winckler, M. Guttman, E. S. Lander, G. Getz, J. P. Mesirov, Integrative genomics viewer. *Nat. Biotechnol.* **29**, 24–26 (2011).

87. O. Pedro, J. A. Mérida-Cerro, A. G. Rondón, B. Gómez-González, A. Aguilera, DNA-RNA hybrids at DSBs interfere with repair by homologous recombination. *eLife* **10**, e69881 (2021).
